# Tumor Microenvironment Characterization Identifies *KIF15* as an Immunosuppressive Driver in Breast Cancer

**DOI:** 10.1155/humu/8861116

**Published:** 2026-01-22

**Authors:** Bo Zhang, Feiran Wang, Huiwei Huang, Xiang Zhong, Qian Chen, Xiancheng Liu

**Affiliations:** ^1^ Research Center of Clinical Medicine, Affiliated Hospital of Nantong University, Nantong, China, ahnmc.com; ^2^ Department of General Surgery, Affiliated Hospital of Nantong University, Nantong, China, ahnmc.com; ^3^ Department of Physiology, Medical College, Nantong University, Nantong, China, ntu.edu.cn; ^4^ Department of Oncology, The Affiliated Suzhou Hospital of Nanjing Medical University, Suzhou Municipal Hospital, Gusu School, Nanjing Medical University, Suzhou, China, njmu.edu.cn; ^5^ Department of Radiation Oncology, Affiliated Hospital of Nantong University, Nantong, Jiangsu, China, ahnmc.com

**Keywords:** breast cancer, immunosuppressive microenvironment, *KIF15*, TMEscore, tumor microenvironment (TME)

## Abstract

The various cellular composition of the tumor microenvironment (TME) comprises the fundamental units of tumor tissue. The types of stromal cells in the TME are genetically stable, with reduced risk of tumor recurrence and drug resistance. More and more evidence shows their clinicopathological significance and therapeutic effect in predicting prognosis. Therefore, we performed an integrated analysis of the breast cancer TME, correlating it with genomic landscapes and clinical profiles. In this work, we first conducted unsupervised hierarchical clustering on 830 tumors in the breast cancer cohort. Then, we defined three TME phenotypes and applied principal component analysis to construct a TMEscore for quantifying TME. Analysis revealed that patients stratified into the high TMEscore cohort exhibited superior survival compared to the low‐scoring group. Additionally, a high TMEscore is associated with an improved response to immunotherapy. Through TME gene signature analysis, *KIF15* was identified as a pivotal driver of the immunosuppressive microenvironment in breast cancer. *KIF15* knockdown may promote dendritic cell infiltration and function, thereby inducing CD8^+^ T cell recruitment. In summary, the immune microenvironment‐derived TMEscore represents an independent prognostic biomarker in breast cancer, while *KIF15* emerges as a crucial molecular determinant of its immunosuppressive niche.

## 1. Introduction

Breast cancer is the most common malignancy in women, representing a leading cause of cancer‐specific mortality [1, 2]. According to the statistics in 2020, about 276,480 women in the United States have been diagnosed with breast cancer, and 42,170 patients are expected to die of cancer [1, 3]. Although over recent years, the breast cancer mortality rate has reduced due to the progress of early identification, accurate diagnosis, and timely treatment, and there are still some BC patients who have obtained adverse results due to different heterogeneity [4, 5].

Cancer arises within complex tissue milieus, which are indispensable for supporting its growth, invasion, and metastatic dissemination. In contrast to malignant cells, stromal cell populations within the tumor microenvironment (TME) are generally genetically stable, making them promising therapeutic targets associated with diminished risks of drug resistance and disease recurrence [6]. Accumulating evidence underscores the pivotal role of the TME in cancer development and treatment outcomes [7, 8]. Immunotherapy, particularly anti‐PD‐1/PD‐L1 therapy, has become a leading modality for treating diverse cancer types [9–11]. However, compared with other cancers, immunotherapy for BRCA has made little progress.

Kinesin family member 15 (*KIF15*), a microtubule‐associated motor protein, mediates mitotic spindle dynamics and chromosome segregation, and its dysregulation has been increasingly implicated in cancer progression. Recent studies demonstrate that *KIF15* overexpression promotes tumor proliferation, invasion, and therapy resistance across various tumor types, including lung, breast, and pancreatic cancer. Nevertheless, the functional significance of KIF15 within the breast TME and its influence on response to immune checkpoint blockade remains unclear [12, 13].

In this study, a comprehensive characterization of TME immune infiltration was conducted by applying CIBERSORT to resolve 22 distinct immune cell fractions. This analysis was performed across a cohort of 8444 breast cancer patients, enabling a systematic investigation of the associations between TME infiltration profiles and clinical outcomes. Based on these findings, we developed a novel metric, the TMEscore, to quantitatively evaluate the TME landscape. The TMEscore demonstrates potential as a robust prognostic indicator and a predictor of responses to immune checkpoint blockade. Moreover, we discovered that KIF15 acts as a pivotal regulator of immunosuppression in breast cancer. It impairs CD8^+^ T cell recruitment by attenuating dendritic cell activity, suggesting that therapeutic targeting of KIF15 could potentially overcome resistance to immunotherapy in this malignancy.

## 2. Materials and Methods

### 2.1. Cells and Animals

The murine breast cancer cell line of 4T1 was procured from the Chinese Academy of Sciences Cell Bank (Shanghai). Cells were maintained in Dulbecco’s modified Eagle’s medium (DMEM) supplemented with 10% fetal bovine serum (FBS) (Gibco, Waltham, Massachusetts, United States) and incubated at 37°C in a humidified incubator with 5% CO₂. A stable *KIF15*‐knockdown cell line was established using shRNA delivered by lentiviral transduction, followed by selection and validation of knockdown efficiency. Female BALB/c mice (aged 6–8 weeks) were sourced from the Laboratory Animal Center of Nantong University and maintained under specific pathogen‐free (SPF) conditions with ad libitum access to food and water. To establish the subcutaneous tumor model, control and KIF15‐knockdown cells were inoculated into the mouse armpit. For immunotherapy, an antimurine PD‐1 monoclonal antibody was obtained from Bio X Cell and administered posttumor engraftment. Following tumor collection, tissues were digested into single‐cell suspensions for subsequent flow cytometric evaluation. All procedures involving animals received approval from the Animal Ethics Committee of Nantong University and were performed following relevant ethical regulations.

### 2.2. Breast Cancer Dataset Collection

We systematically retrieved publicly accessible transcriptomic datasets with complete clinical metadata from the Gene Expression Omnibus (GEO) and The Cancer Genome Atlas (TCGA) repositories. Our dataset curation process identified 48 eligible breast cancer cohorts, following the exclusion of cases lacking survival data: In these datasets,16 datasets had overall survival (OS) data, 6 datasets had DFS data, 1 dataset with DRFS data, and 1 dataset with DMFS. All microarray datasets were downloaded from GEO background correction, and quantile normalization and probe summarization were conducted with the RMA algorithm, which applies a median polish procedure for final expression estimation. For TCGA cohorts, transcriptomic data in FPKM format were acquired via the R package TCGAbiolinks based on the GDC portal [14]. These values were subsequently converted to transcripts per kilobase million (TPM) for downstream analyses [15]. To account for potential batch effects arising from merging multiple gene expression datasets, we performed batch correction using the ComBat function from the sva R package (v3.46.0). Briefly, after ensuring that all datasets shared a common set of genes and removing duplicate gene entries, we combined the expression matrices into a single matrix. The batch variable was defined based on the original dataset source for each sample. We applied ComBat(dat = as.matrix(expr_all), batch = batch, par.prior = TRUE, prior.plots = FALSE), where par.prior = TRUE enables empirical Bayes parameter estimation, improving stability for datasets with small sample sizes. Data were analyzed with the R (Version 4.1.2) and R Bioconductor packages. All datasets were public, deidentified, and did not require fresh consent.

### 2.3. Acquisition of Clinical Annotations for Breast Cancer Cohorts

For GEO series, clinical metadata absent from expression profiles were sourced through the following approaches: (i) retrieval from the respective dataset page on the GEO platform, (ii) extraction from supporting files of associated publications, or (iii) utilization of the GEOquery package in R. Regarding TCGA cohort, clinical attributes and sample details for TCGA‐BRCA project were accessed via TCGAbiolinks R package [14] from the Genomic Data Commons [14]. OS data across TCGA cohorts were derived from a previously published resource [16]. Somatic mutation profiles, including single‐nucleotide polymorphisms and short insertions/deletions processed by MuTect2 Variant Aggregation and Masking, for patients with breast cancer were acquired from TCGA repository.

### 2.4. Calculation of Infiltrating Cells in the TME

The proportions of immune cells in breast cancer samples were quantified using the CIBERSORT algorithm [12], and the LM22 signature, a method capable of sensitively and specifically discriminating 22 human immune cell phenotypes, including macrophages, natural killer cells, B cells, T cells, DCs, and myeloid subsets. CIBERSORT applies a support vector regression machine learning framework to assess the relative fractions of distinct cell types within heterogeneous tissue samples. This computational approach relies on a predefined signature matrix (LM22) containing 547 genes that uniquely characterize 22 immune cell phenotypes. For our analysis, normalized gene expression data were annotated using standard platforms and subsequently processed through the CIBERSORT online tool, configured with the LM22 signature and 1000 permutation iterations.

### 2.5. Identification of TME Infiltration Patterns Through Unsupervised Clustering

To categorize tumors based on their immune cell infiltration profiles, we performed hierarchical agglomerative clustering with Euclidean distance and Ward’s linkage (ward.D). Patterns within the TME were further characterized using K‐means clustering, which grouped patients into distinct subsets for subsequent analyses. To evaluate the robustness of these clusters across multiple GEO datasets, a consensus clustering approach was implemented with the R package ConsensusClusterPlus [17]. This process involved 1000 iterative runs to confirm classification stability [17].

### 2.6. Calculation of Differentially Expressed Genes (DEGs) and Dimension Reduction of Distinct TME Groups

To uncover genes linked to TME infiltration patterns, patients were first stratified into distinct groups according to immune cell composition. Differential expression analysis across these subgroups was conducted with the limma package [18], applying an adjusted *p* value threshold of < 0.05 after Benjamini–Hochberg false discovery rate (FDR) correction. Technical batch effects were mitigated using the sva package [19]. Subsequently, all significant genes were *z*‐score normalized across integrated GEO datasets. These standardized genes then underwent K‐means clustering to categorize patients for downstream investigation. To refine the feature set and minimize noise, a random forest‐based classifier was employed for dimensionality reduction. Functional annotation of resulting gene modules was performed with clusterProfiler, while consensus clustering helped establish robust gene classifications [20].

### 2.7. Calculation of TME Score

After the identification of TME signature genes, gene expression values within each signature were standardized by *z*‐score transformation. PCA was conducted, and the PC1 and PC2 two leading components were retained as signature scores. To compute the TMEscore for each patient, we adapted an approach previously described for the GGI [21]. The formula applied was TMEscore = *Σ*(PC1_*i*_ + PC2_*i*_) − *Σ*(PC1_*j*_ + PC2_*j*_), where *i* denotes gene clusters associated with a positive Cox coefficient and *j* indicates those showing a negative Cox coefficient.

### 2.8. Functional Enrichment Analysis

The GO and KEGG pathway analyses were carried out with the clusterProfiler package, aimed at characterizing the biological functions of the TME‐related signature genes, retaining terms that passed a significance threshold of *p* < 0.05 [20]. GSEA was further applied to compare TME gene Groups 1 and 2 under specific phenotypic contexts, using preprocessed expression profiles of all transcripts [22]. Reference gene sets were acquired from the Broad Institute’s MSigDB repository, with HALLMARK collections employed to evaluate pathway activity. All enrichment results were generated based on 1000 permutation tests and adjusted via the Benjamini–Hochberg method for FDR control. Result visualization was facilitated by the enrichplot R package, which supports intuitive interpretation of GSEA outcomes.

### 2.9. Association of TME Signatures With Biological Processes

To investigate relationships between the TME signature and key cancer‐related pathways, we utilized gene sets previously established by Mariathasan et al. [23]. These comprised the following: (1) a CD8^+^ T cell effector signature [24], (2) antigen presentation machinery genes [25], (3) immune checkpoint molecules, (4) published epithelial–mesenchymal transition markers (EMT1–3) [26], (5) a pan‐fibroblast TGF‐*β* response signature (Pan‐F‐TBRS) [23], (6) a previously defined angiogenesis signature [27], (7) Fanconi anemia pathway genes, and (8) KEGG pathways covering cell cycle regulation, mismatch repair, homologous recombination, nucleotide excision repair, DNA replication, DNA damage repair, and WNT targets.

### 2.10. Validation Using Immune Checkpoint Blockade Dataset

We evaluated the predictive utility of the TMEscore using transcriptomic data from individuals with metastatic urothelial cancer who received the anti‐PD‐L1 therapy mepolizumab [24]. The IMvigor210 cohort data, including comprehensively annotated clinical and molecular profiles, is accessible under the Creative Commons 3.0 license. The complete analysis package can be retrieved from http://research-pub.gene.com/IMvigor210CoreBiologies.

### 2.11. Statistical Analysis

Group comparisons were performed using the Wilcoxon rank‐sum test. Correlations were assessed through Spearman and distance correlation methods. Categorical data were evaluated with two‐sided Fisher’s exact tests [28]. Optimal TMEscore thresholds for each dataset were determined by analyzing OS associations via the survminer package. The MaxStat package implemented iterative cutpoint testing to dichotomize TMEscore into low and high categories based on maximum rank statistics. Subgroup analyses across breast cancer cohorts were visualized using the forestplot package. In differential expression analysis, FDRs were derived from *p* values using the Benjamini–Hochberg procedure. Survival curves generated by the Kaplan–Meier method were compared with log‐rank tests. Hazard ratios were computed through univariate Cox regression, while multivariate Cox models identified independent prognostic factors. Diagnostic performance of tumor mutational burden (TMB), TMEscore, and their combination was quantified by area under the curve (AUC) metrics from ROC curves generated with pROC [29]. AUC comparisons used likelihood ratio tests for correlated curves. Phylogenetic relationships among TME signature genes were visualized with ggtree [30], and heat maps were created using the pheatmap function. A two‐sided *p* value < 0.05 was adopted as the threshold for statistical significance.

## 3. Results

### 3.1. Breast Cancer TME Overview

Firstly, 81 datasets with a sample size of over 50 were finally screened out from the GEO database (Figure 1a). Next, the datasets with expressed genes of less than 10,000 were removed. Finally, 48 datasets with 8444 cases in total were enrolled in the CIBERSORT analysis. After removing duplicate samples, 7 datasets with available OS data and clinical information were enrolled into one metacohort (GSE16446, *n* = 30, GSE18229, *n* = 196, GSE20624, *n* = 290, GSE20711, *n* = 29, GSE26304, *n* = 104, GSE42568, *n* = 88, and GSE6130, *n* = 93), which were treated as a training cohort (Table S1). PCA showed that the batch effect of these datasets was eliminated (Figure S1A). Totally, there were 295 ER‐positive and 184 ER‐negative samples in the training set. The TME cellular network elucidated the complex interplay between tumor and immune components, along with distinct cell lineage relationships, delineating their collective impact on breast cancer patient survival (Figure 1b). The TME cellular network revealed that neutrophils, macrophages, plasma cells, and Treg cells were significantly related to poor patient prognosis, whereas dendritic cells, monocytes, mast cells, and B cells correlated with markedly prolonged survival. Moreover, we found that close interactions exist among cells within the TME, which may mediate treatment response and patient survival.

Figure 1Global view of study design and TME subtypes. (a) Overall design and workflow of the study. (b) Associations of TME cell types. Cellular interactions of Cell Clusters A, B, C, and D are highlighted with blue, cyan, brown, magenta, and yellow, respectively. The node size corresponds to −log_10_(*p* value) of the significance between each TME cell type and patient survival. The positive/negative correlations between each node are colored in red/blue, and the line width corresponds to the strength of the correlation value generated by the Spearman correlation test. Favorable and risk factors are indicated in green and black.

**Figure:**
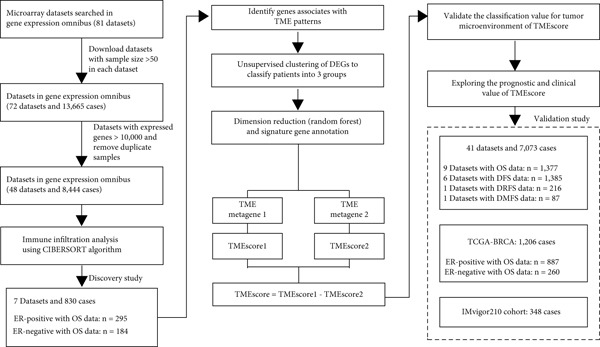
(a)

**Figure:**
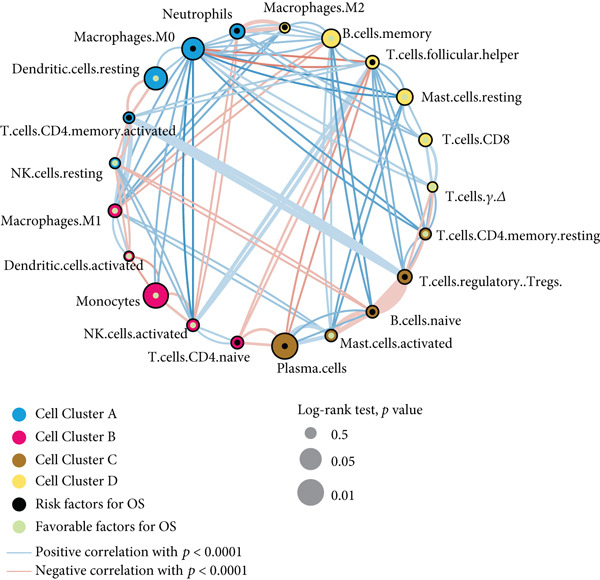
(b)

### 3.2. Unsupervised Hierarchical Clustering of TME Cell

Patient stratification was performed via unsupervised hierarchical clustering according to the abundances of 22 immune cell types to explore the heterogeneity and clinical significance of the breast cancer TME. The analysis encompassed 830 tumors from independent breast cancer cohorts with available TME expression data (Figure 2a). As shown in the results, unsupervised clustering identified three TME subgroups with the following distribution: TMEgroup‐A (*n* = 316), TMEgroup‐B (*n* = 233), and TMEgroup‐C (*n* = 281) (Figure 2a and Figure S1B). The associations between the three TME groups and patient survival and the result indicated that the OS was significantly different in these TME groups (Figure 2b, *p* value < 0.001). Notably, TME Cluster B demonstrated significantly prolonged survival, whereas TME Cluster A exhibited the poorest clinical outcomes. Next, the main immune cells within the three TME groups were subsequently analyzed, and the result showed that in TMEgroup‐A, the immune cells were T.cells.follicular.helper, Macrophages.M0, and Plasma.cells., and the main immune cells in TMEgroup‐B were Macrophages.M2, T.cells.CD4.memory.resting, Mast.cells.resting, and monocytes, whereas the main immune cells in TMEgroup‐C were T.cells.CD8, B.cells.memory, Macrophages.M1, Tregs, and NK.cells.activated (Figure 2c).

Figure 2Characterization of TME clusters. (a) Unsupervised hierarchical clustering of TME cells. Patient information, including the GEO dataset, survival status, PR status, ER status, HER2 status, and tumor stages, is all listed at the top of the heat map. (b) KM plot for overall survival of patients. Patients in different TME groups are marked with different colors. (c) Comparisons of TME cell composition among three TME groups using boxplots.  ^∗^ *p* < 0.05;  ^∗∗^ *p* < 0.01;  ^∗∗∗^ *p* < 0.001;  ^∗∗∗∗^ *p* < 0.0001.

**Figure:**
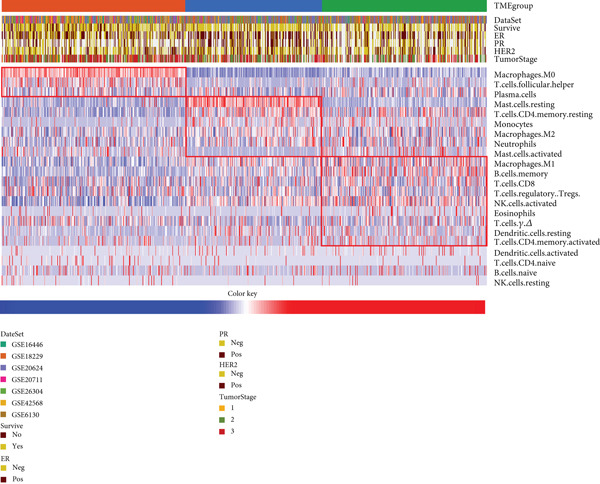
(a)

**Figure:**
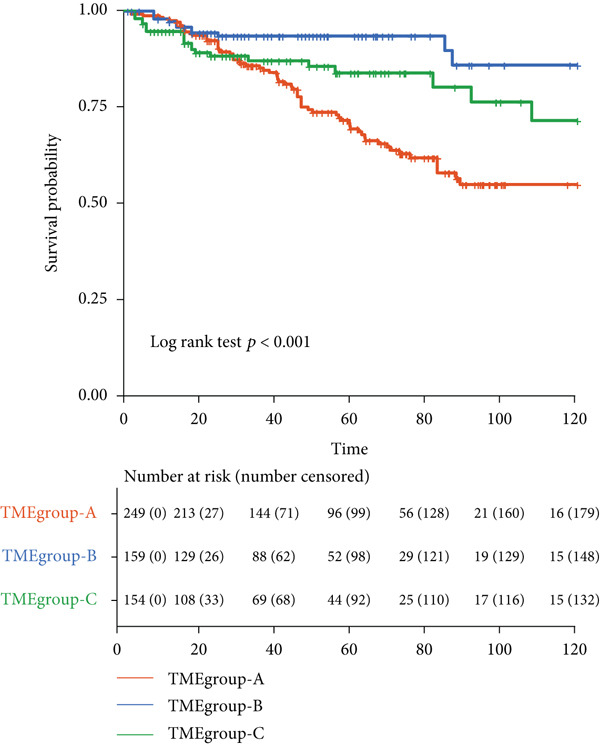
(b)

**Figure:**
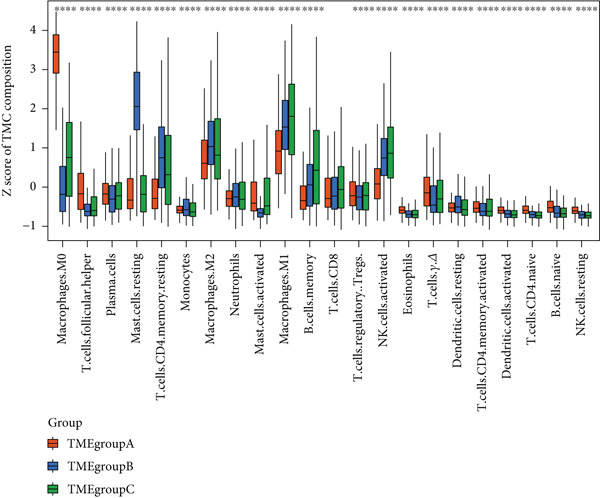
(c)

### 3.3. TME Signature and Gene Functions

Unsupervised clustering of the TME cellular landscape revealed three breast cancer subtypes with distinct prognostic profiles. Differential gene expression analysis of these subtypes identified 53 consistently dysregulated genes across all subgroups, uncovering their unique molecular signatures (Figure S1C,D). Following dimensionality reduction, phenotypic signatures were derived from the processed data. Four hundred and sixty‐one genes were identified to unsupervised cluster the 830 tumors into two groups based on gene expression profile (Figure 3a and Figure S2A). TME Signature Gene 1 contains 190 genes, and TME Signature Gene 2 contains 271 genes (Figure 3a and Figure S2B). And the OS in TME Gene Groups A and B was significantly different (Figure 3b). Genes in TME Gene Group‐A were enriched in NF‐kappa B signaling pathway, cell cycle, rheumatoid arthritis, microRNAs in cancer, DNA replication, primary immunodeficiency, Fc gamma R‐mediated phagocytosis, oocyte meiosis, homologous recombination, and bladder cancer (Figure 3c). Genes in TME Gene Group‐B were PI3K‐Akt signaling pathway, ECM‐receptor interaction, enriched in focal adhesion, proteoglycans in cancer, protein digestion and absorption, and human papillomavirus infection (Figure 3d). Macrophages.M0 cells clustered predominantly in TME Gene Group‐A, in contrast to mast cells resting, which were primarily found in Group‐B (Figure 3e). The common DEGs in TME Gene Group‐A and TME Gene Group‐B were enriched in triple‐negative breast neoplasms and mammary neoplasms with enrichment in the disease‐related database (Figure S2C). Immune‐related genes CD8A, IFNG, PRF1, TNF, AVCR2, LAG3, PDCD1, COL4A1, SMAD9, TWIST1, and ZEB1 were highly expressed in the TME Gene Group‐A (Figures S3A, S3B, and S3C). And the correlation between Pan‐F‐TBRS, homologous recombination, DNA replication, mismatch repair, DNA damage repair, cell cycle, and TMEscore2 was positive, whereas CD8 T effector, Fanconi anemia, and TMEscore1 were positive (Figure S3D). TMEscore was relatively independent in the multivariate analysis (Figure S3E).

Figure 3TME gene signatures and functions. (a) Unsupervised hierarchical clustering of DEGs. Gene groups, TME groups, patient dataset, survival status, ER status, PR status, HER2 status, and tumor stages are also listed above the heat map. (b) KM plot of patient groups, TME Gene Group‐A and ‐B are colored with red and blue, respectively. (c, d) KEGG analysis of genes involved with two gene signatures. The *x*‐axis and bar height correspond to −log_10_(*p* value). (e) Comparisons of TME cell composition between two gene groups using boxplots.

**Figure:**
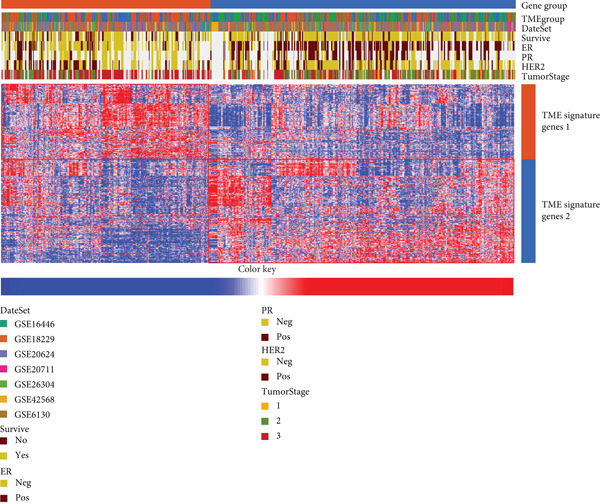
(a)

**Figure:**
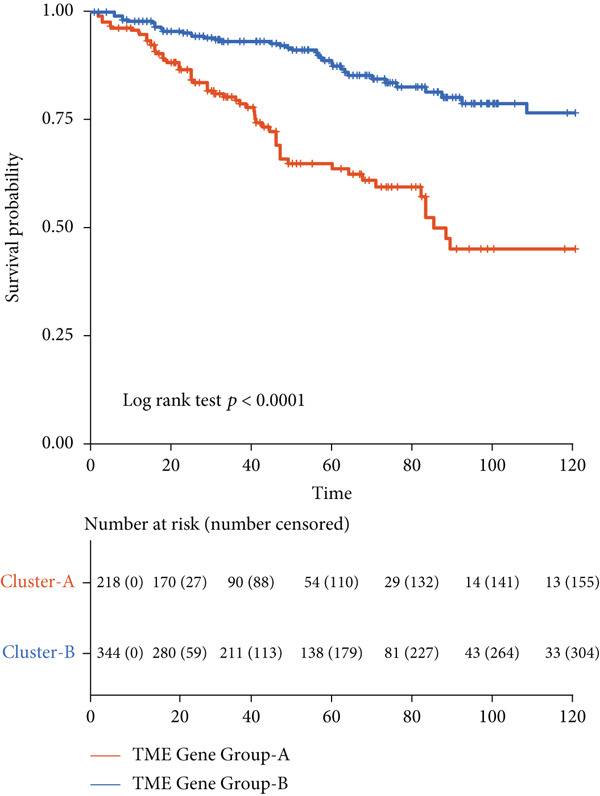
(b)

**Figure:**
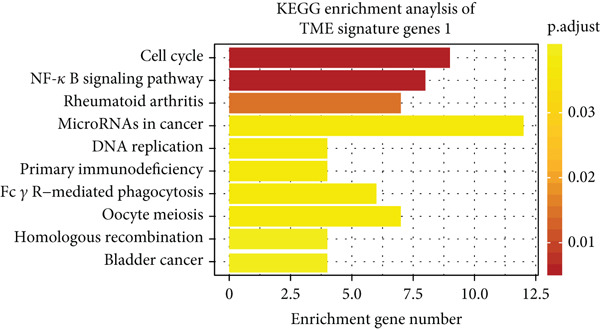
(c)

**Figure:**
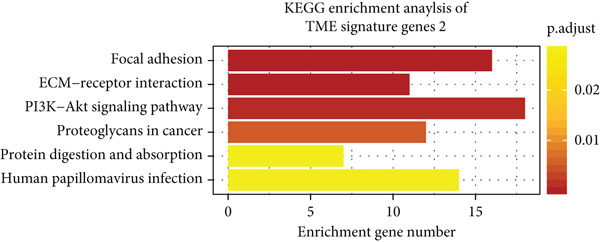
(d)

**Figure:**
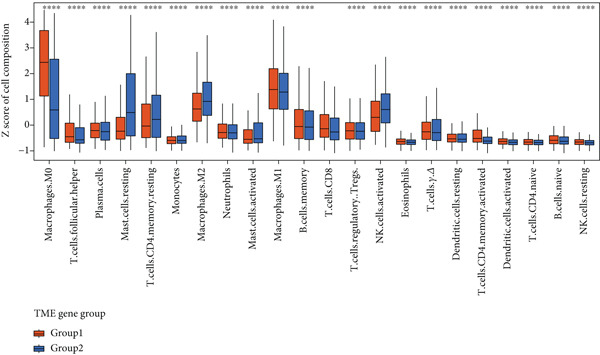
(e)

### 3.4. TMEscore Construction and Clinical Significance

We use the PCA algorithm to construct the TMEscore based on the TME Signatures A and B to evaluate its role in breast cancer. We found that the patients with a higher TMEscore experienced a prolonged survival than those with a lower TMEscore (Figure 4a). The TMEscore as well as TMEscores A and B showed a significant difference between the three TME groups among the different cohorts (Figures S4 and S5). Furthermore, the TMEscore exhibited significantly higher expression levels in ER‐positive and HER2‐positive cases, while showing no association with PR status (Figure 4b). Among different molecular subtypes of breast cancer, HER2‐enriched and Luminal B subtypes exhibited the highest TMEscores (Figure 4c), while elevated TMEscores were significantly associated with increased TMB (Figure 4d). EMT2, Pan‐F‐TBRS, CD8.T.effector, DNA.replication, DNA.damage.repair, Immune.checkpoint, Nucleotide.excision.repair, EMT3, Antigen.processing.machiner, and Cell.cycle.regulators were positively correlated with TMEscore2, and WNT.target, EMT‐1, Homologous.recombination, Fanconi.anemia, Cell.cycle, Mismatch.repair, and angiogenesis were positively correlated with TMEscore (Figure S6A). The GEO cohort independently confirmed the prognostic capacity of the TMEscore (Figure 4e and Figures S6B and S7A, S7B, S7C, S7D, S7E, and S7F).

Figure 4Characterization of TMEscore within different cohorts. (a) KM plot of patients with different TMEscore. (b) Comparison of TME scores within different clinical groups referring to ER, HER2, and PR expression. *p* values are marked at the boxplot top. (c) Comparison of TMEscore within different kinds of patients. *p* values are marked at the top of the boxplots. (d) Tumor burden comparison between patients with high/low TMEscore. (e) Forest plot of clinical prognostic values between high/low TMEscore patients. The line represents 95% CI for each group, and the dotted line represents the HR of all patients. The number of patients is indicated with the area of gray rectangles.

**Figure:**
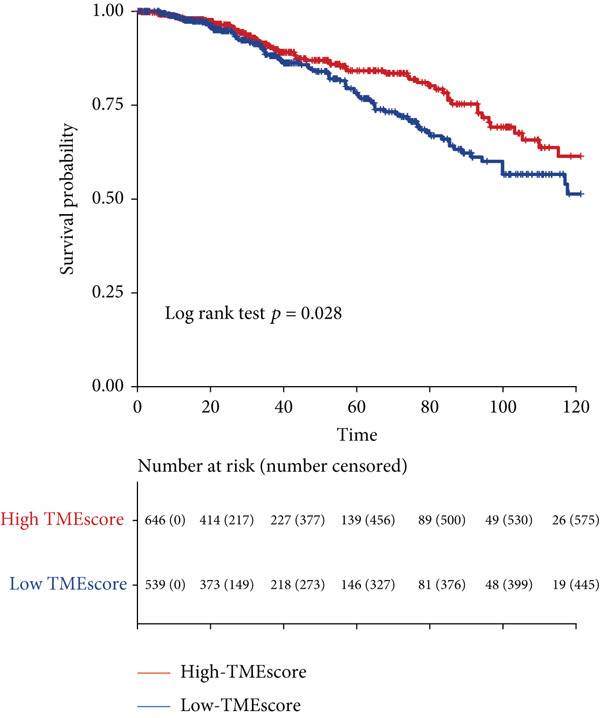
(a)

**Figure:**
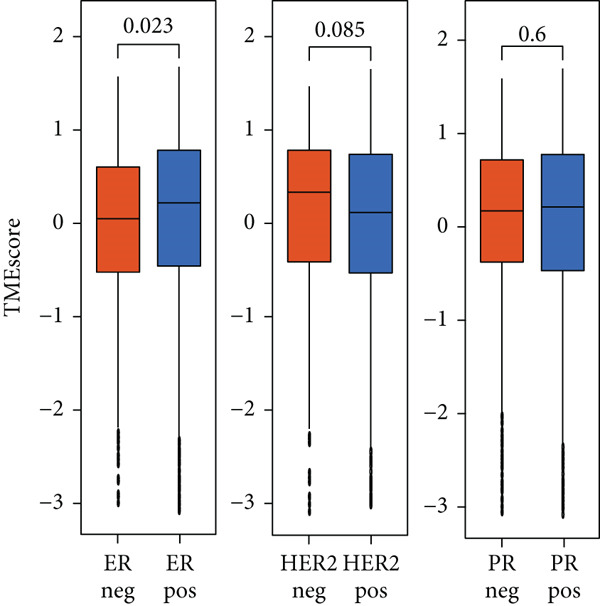
(b)

**Figure:**
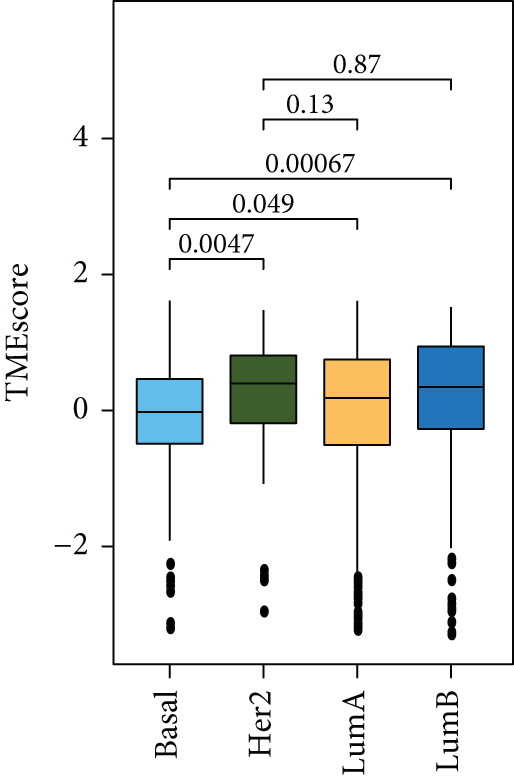
(c)

**Figure:**
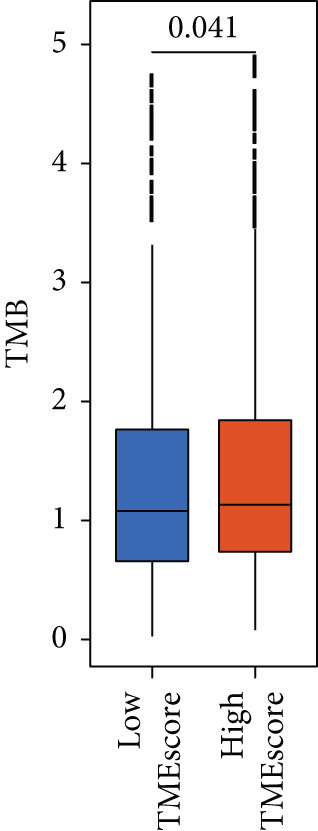
(d)

**Figure:**
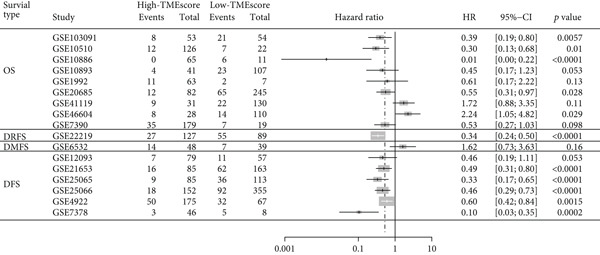
(e)

### 3.5. TMEscore in Predicting Immunotherapy Response

To validate the relationship between immune cells, TMEscores, and TMB, we use the IMvigor210 dataset, which investigated the efficacy of PD‐1 immunotherapy, as the test cohort. Samples that had higher TMEscore also had a better OS (Figure 5a and Figure S8A,B), whereas the prognostic effect was the worst in samples with lower TMEscore and TMB (Figure 5b). Samples after CR/PR were more easily grouped into the higher TMEscore. And the TMEscore in PD is the lowest (Figure 5c,d and Figure S8C). The prediction effect after a combination of TMEscore and TMB was the best (Figure 5e,f). WNT.target, EMT2, EMT‐1, Nucleotide.excision.repair, Pan‐F‐TBRS, DNA.replication, angiogenesis, Immune.checkpoint, Mismatch.repair, and Fanconi.anemia were positively correlated to TMEscore1, and EMT2, EMT‐1, and Pan‐F‐TBRS were positively correlated to TMEscore2 in IMvigor210 cohort (Figure S8D).

Figure 5Clinical significance of TMEscore. (a) KM plot of patients with high and low TMEscore in IMvigor210 dataset. Patients with high and low TMEscore are colored in red and blue. *p* value (log‐rank test) is marked in the plot. (b) KM plot of patients with different values of TMEscore and TMB in the IMvigor210 cohort. Patient groups (HH, HL, LH, and LL) are color‐coded as red, blue, green, and yellow, respectively, as indicated. (c) Comparison of clinical response (CR/PR vs. SD/PD) between low and high TMEscore groups. (d) Comparison of TMEscore within CR, PD, PR, and SD patients. *p* values are marked at the top. (e) ROC curve of TMB, TMEscore, and TMB + TMEscore in IMvigor210 set. The AUC values are also labeled in the plot. (f) ROC curve of TMEscore in TCGA BRCA cohort.

**Figure:**
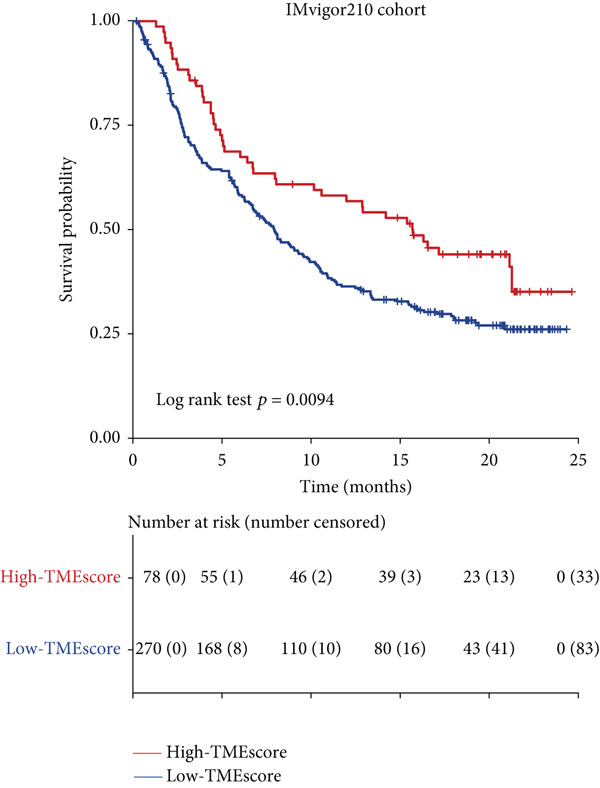
(a)

**Figure:**
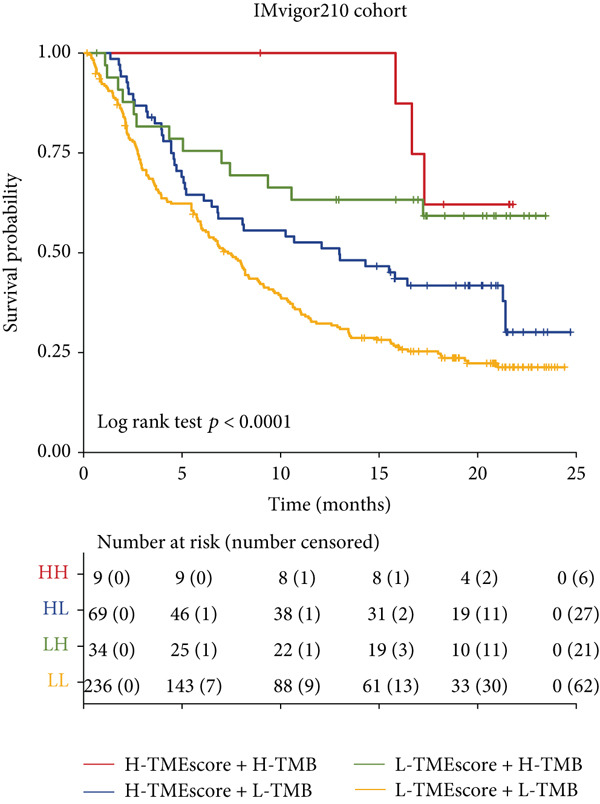
(b)

**Figure:**
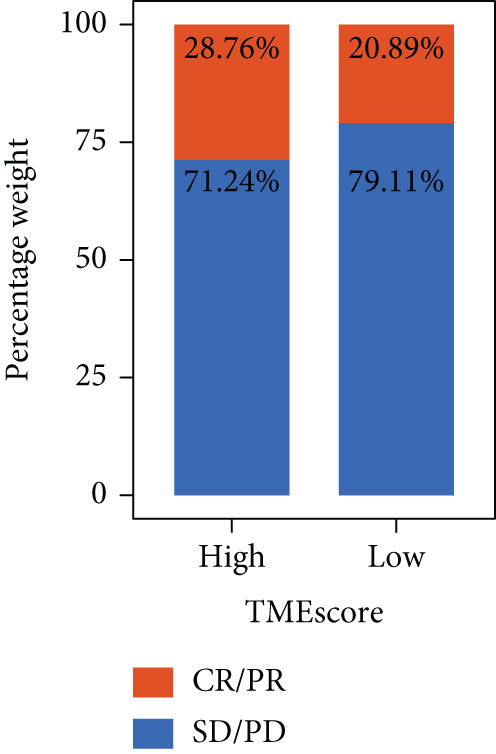
(c)

**Figure:**
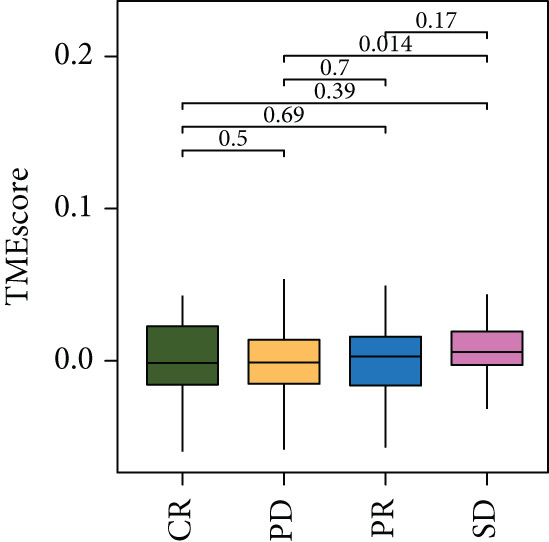
(d)

**Figure:**
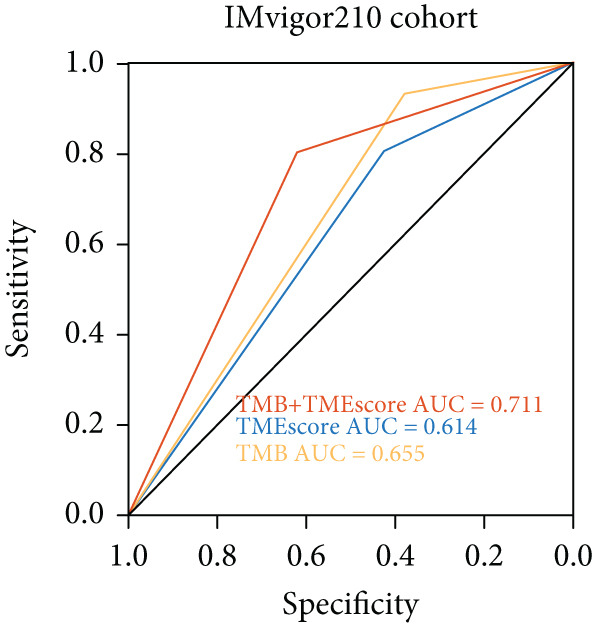
(e)

**Figure:**
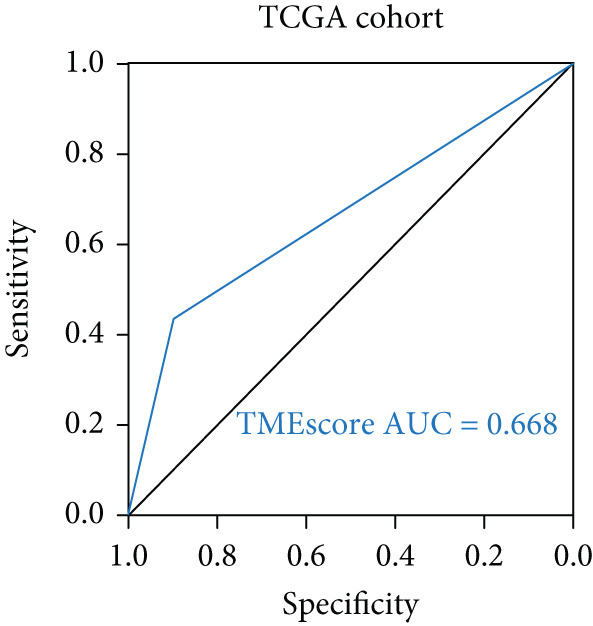
(f)

### 3.6. TME Gene Signature Identifies KIF15 as a Key Driver in Breast Cancer

Based on a gene signature derived from the TMEscore, we identified *KIF15* as a key contributor in model construction. Analysis of multiple breast cancer cohorts from TCGA and GEO consistently revealed elevated KIF15 expression in tumor tissues (Figure 6a). Notably, *KIF15* expression was significantly lower in PR‐ and ER‐positive samples, whereas no significant differences were observed in HER2‐positive or HER2‐negative samples. The relationship between KIF15 levels and immune infiltration was evaluated with CIBERSORT and ESTIMATE algorithms (Figure 6b). Analysis of multiple GEO cohorts revealed that patients with low *KIF15* expression exhibited significantly prolonged survival (Figure 6c). Furthermore, we assessed the correlation between KIF15 expression and a spectrum of immune‐regulatory molecules, including activators, inhibitors, and antigen‐presenting machinery (Figure 6d). Notably, cases with high *KIF15* expression showed a significantly increased probability of TP53 mutation (Figure 6e). GO, KEGG, and GSEA enrichment analyses further demonstrated that *KIF15* is primarily associated with immune and metabolic pathways (Figure 6f,g).

Figure 6Prognostic and functional analysis of *KIF15* in breast cancer. (a) *KIF15* expression between normal and tumor tissues, as well as among different HER2, PR, and ER status groups. (b) Correlation analysis between immune cell abundance and *KIF15* expression across different cohorts. (c) Prognostic analysis of *KIF15* in various cohorts. (d) Correlation between *KIF15* expression and immune inhibitory genes, immune‐activating genes, and antigen presentation‐related genes. (e) Gene mutation analysis in *KIF15* low and high expression groups. (f) GO analysis of *KIF15*. (g) KEGG functional enrichment analysis of *KIF15*. (h) GSEA functional enrichment analysis of *KIF15*.

**Figure:**
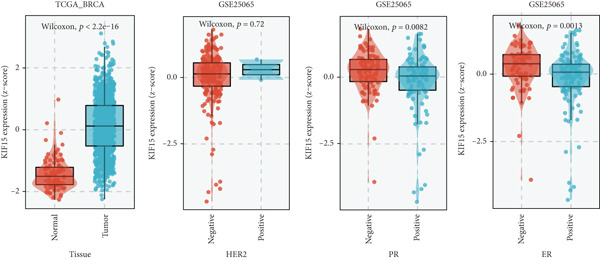
(a)

**Figure:**
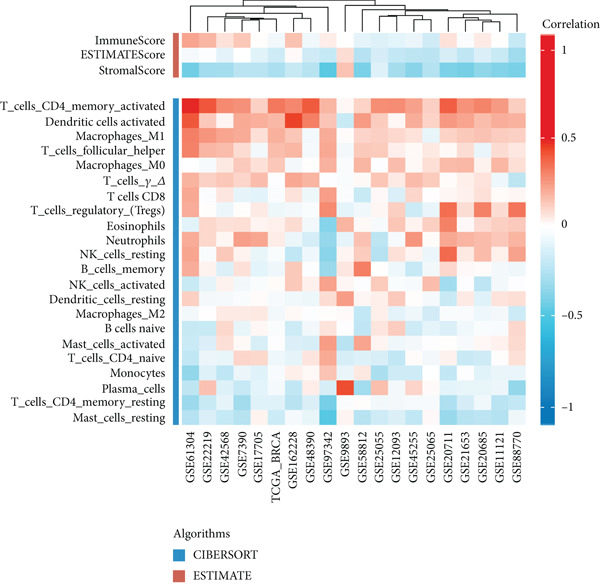
(b)

**Figure:**
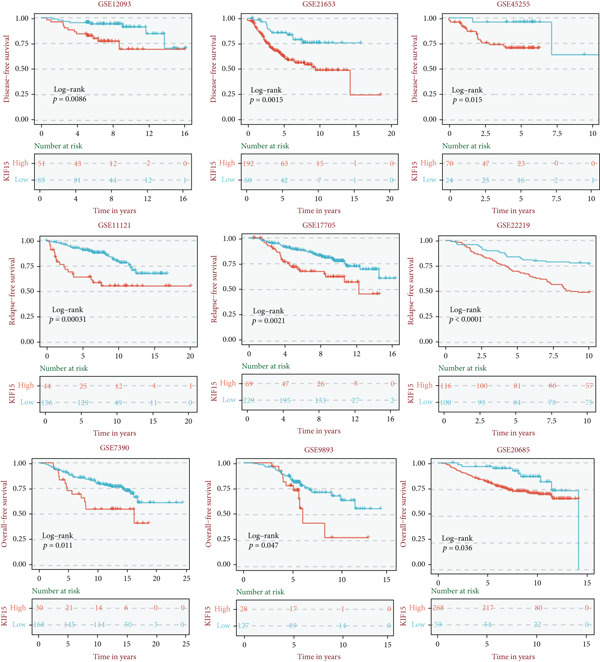
(c)

**Figure:**
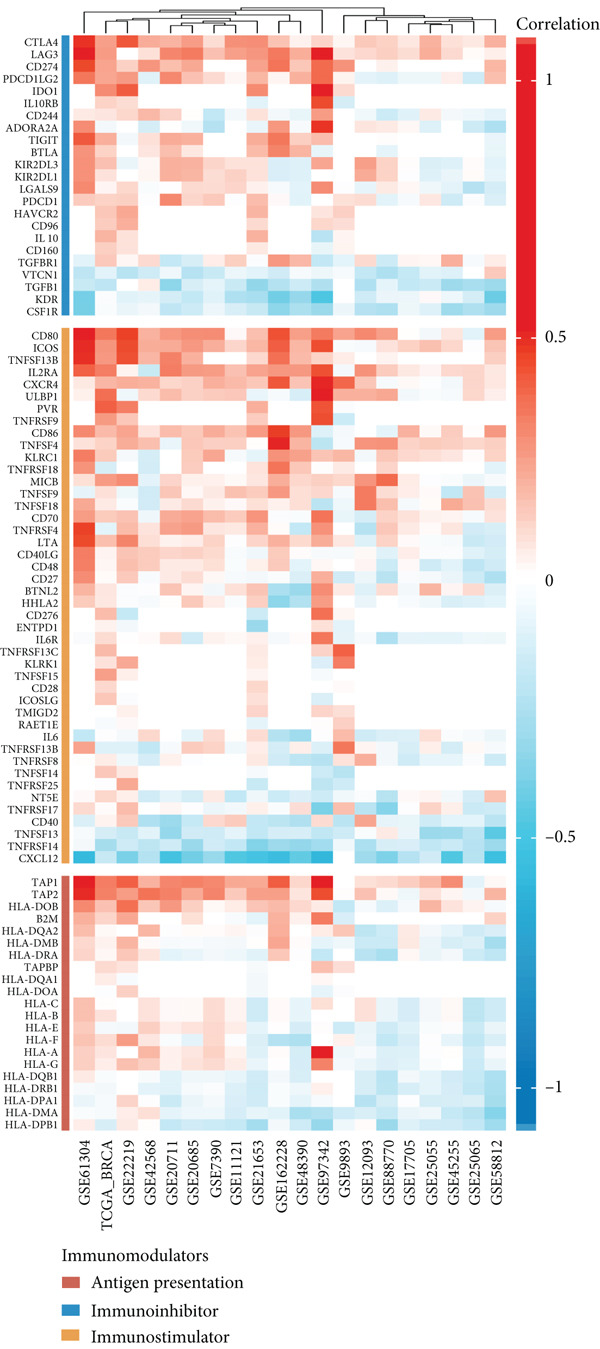
(d)

**Figure:**
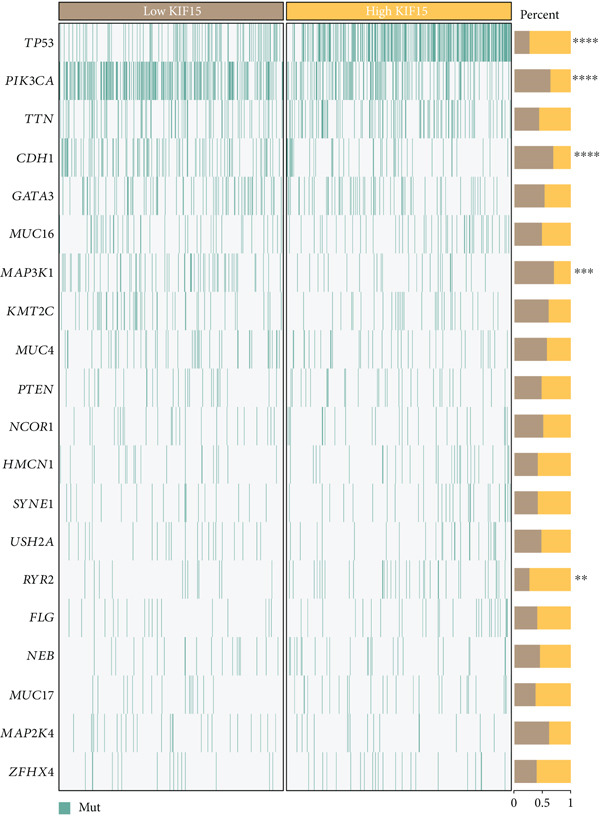
(e)

**Figure:**
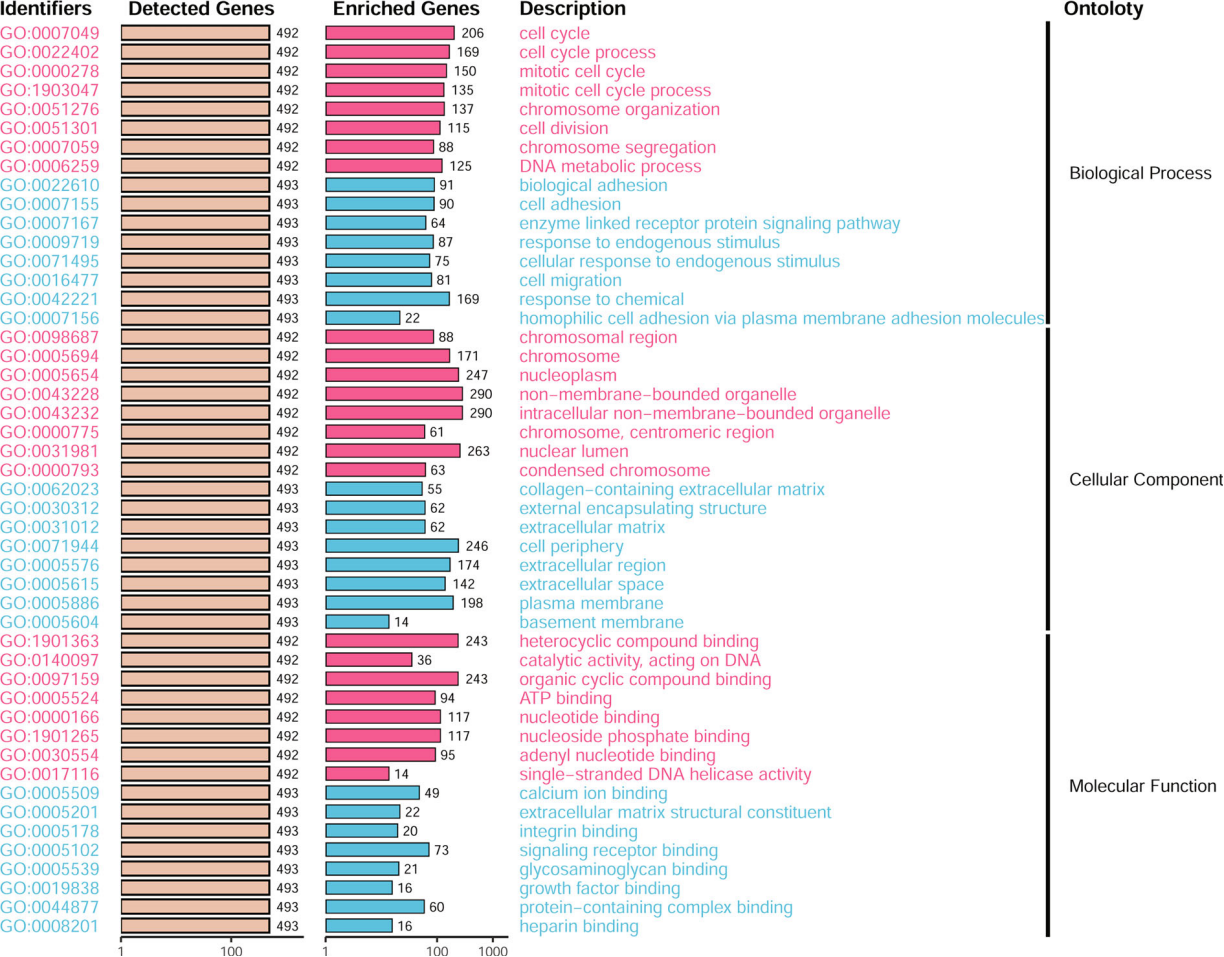
(f)

**Figure:**
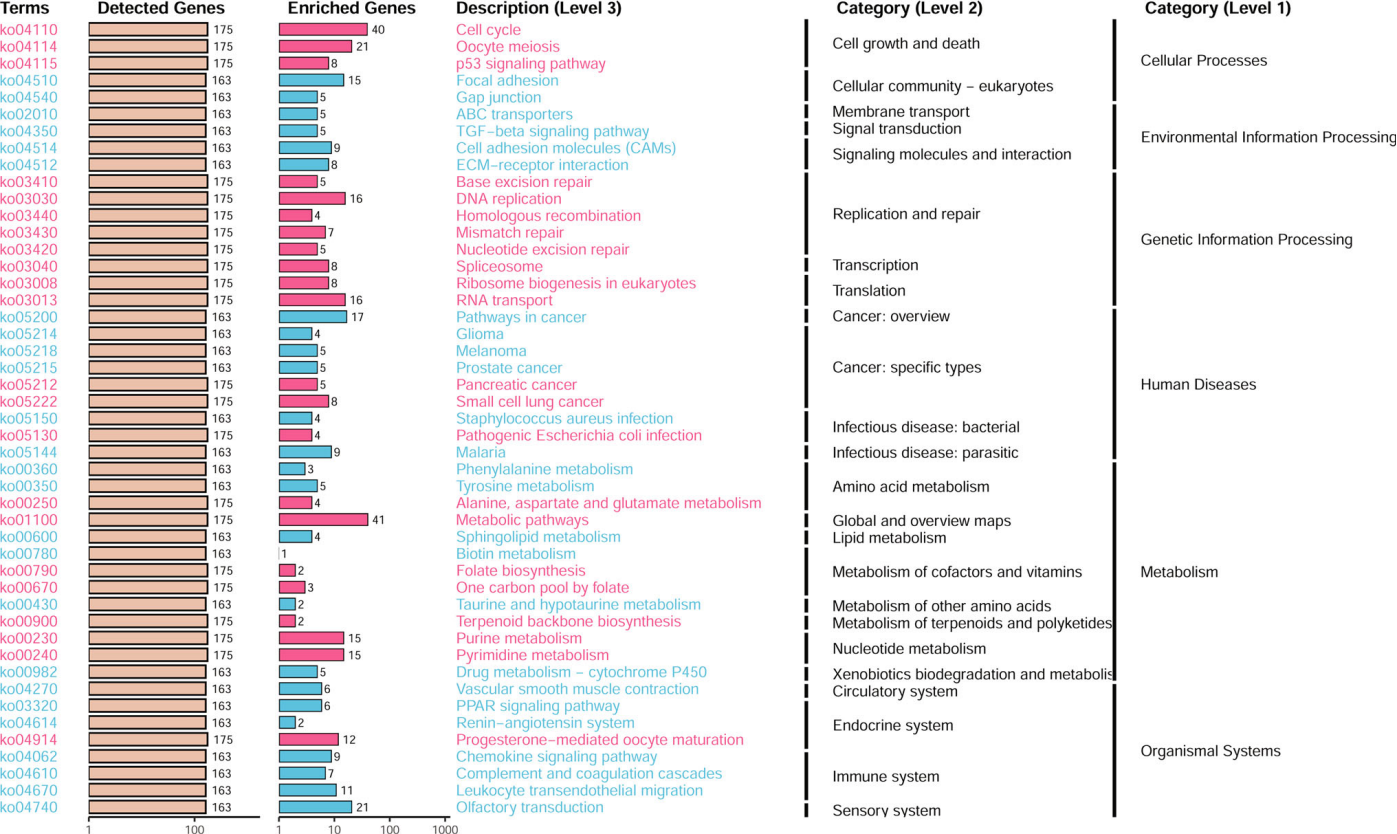
(g)

**Figure:**
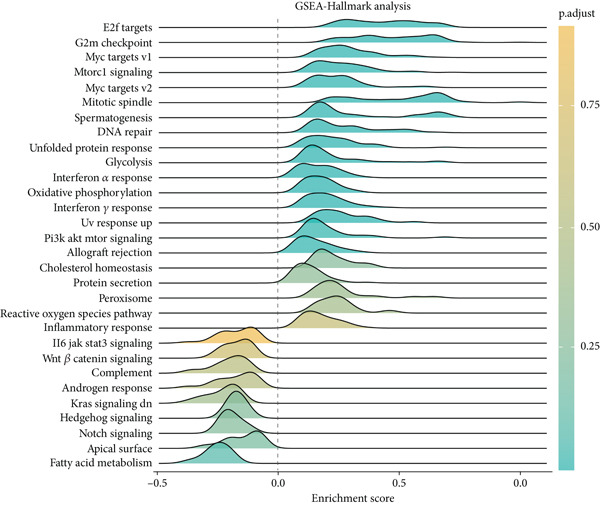
(h)

### 3.7. KIF15 Promotes an Immunosuppressive TME in Breast Cancer

To further investigate the influence of *KIF15* on the TME and immunotherapy response in breast cancer, we generated a *KIF15*‐knockdown murine breast cancer cell line. The results demonstrated that *KIF15* knockdown significantly suppressed tumor growth and enhanced the efficacy of immunotherapy (Figure 7a,b). Tumor tissues were harvested from mice, digested into single‐cell suspensions, and analyzed by flow cytometry. The findings revealed that *KIF15* inhibition markedly promoted CD8^+^ T cell infiltration without affecting expression of its exhaustion marker PD‐1 (Figure 7c,d). In the myeloid immune cell panel, *KIF15* knockdown had no significant impact on macrophage infiltration or polarization but significantly promoted dendritic cell infiltration (Figures 8a,b). Therefore, we hypothesized that *KIF15* could mediate the immunosuppressive TME in breast cancer by inhibiting dendritic cell infiltration.

Figure 7
*KIF15* knockdown suppresses tumor progression and potentiates anti‐PD‐1 immunotherapy. (a) KIF15 knockdown potently potentiates the therapeutic activity of anti‐PD‐1 treatment (*n* = 5). (b) Comparison of tumor weight differences among different treatment groups. Tumor weight comparisons between groups were analyzed using one‐way ANOVA, followed by Tukey’s post hoc test for multiple comparisons. (c) *KIF15* knockdown promotes CD8^+^ T cell infiltration. (d) Comparative analysis of immune infiltration in between the KIF15 knockdown and control groups.

**Figure:**
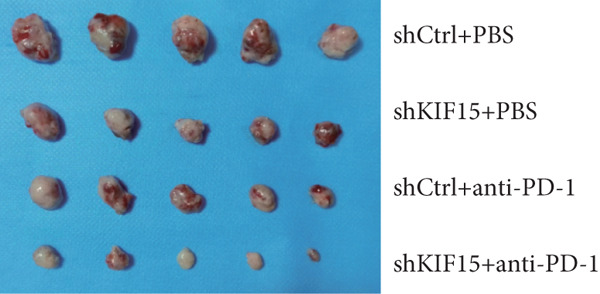
(a)

**Figure:**
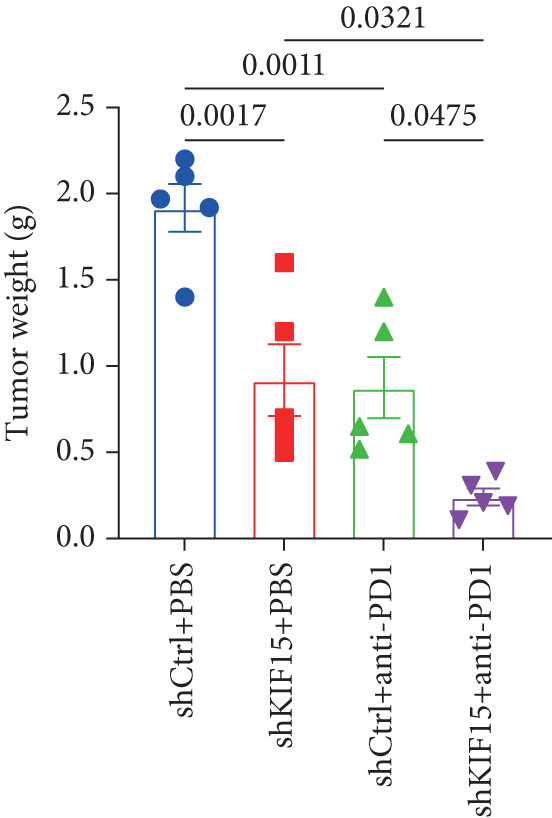
(b)

**Figure:**
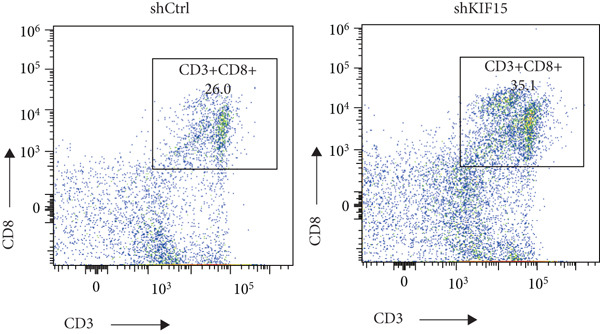
(c)

**Figure:**
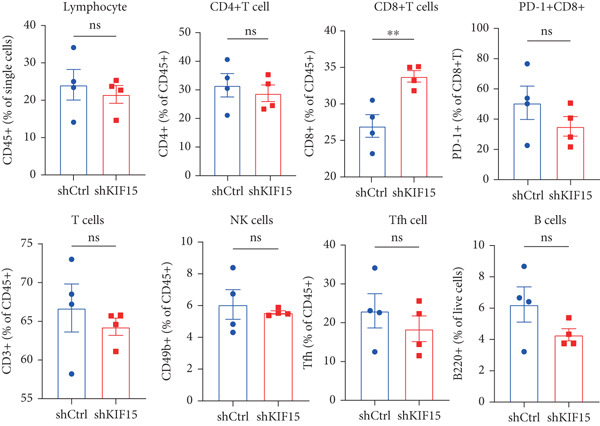
(d)

Figure 8
*KIF15* knockdown promotes dendritic cell infiltration and maturation. (a) *KIF15* knockdown has no significant effect on macrophage infiltration and polarization. (b) *KIF15* knockdown enhances dendritic cell infiltration and maturation.

**Figure:**
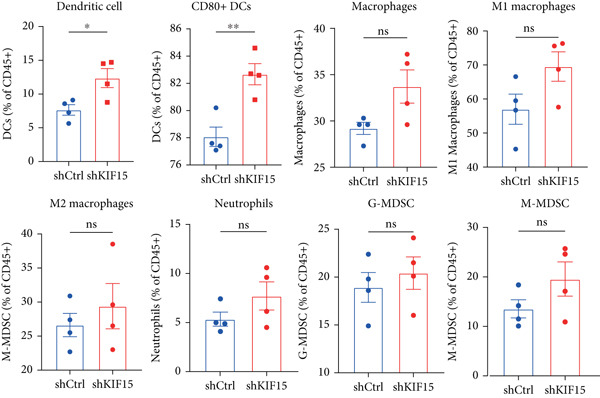
(a)

**Figure:**
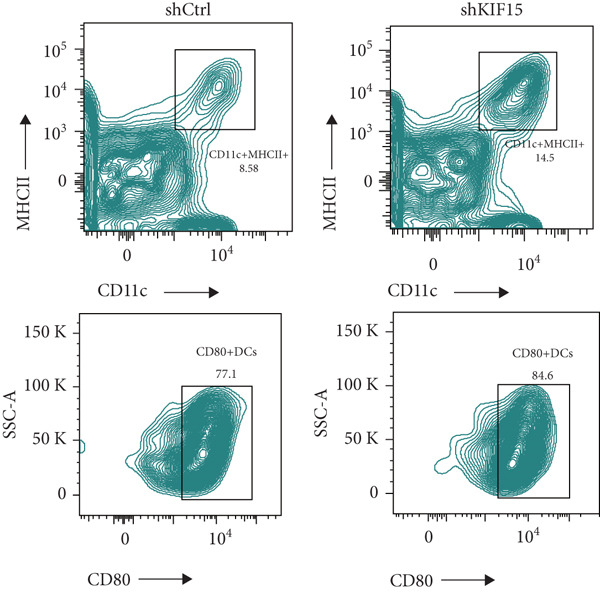
(b)

## 4. Discussion

Breast cancer exhibits significant heterogeneity, and its complex TME notably contributes to variable therapeutic outcomes [31]. Multiple cellular components constitute the TME, such as immune and stromal cells, malignant cells, and cancer‐associated fibroblasts [32]. Accumulating evidence indicates a central role for the TME in regulating breast cancer progression and treatment efficacy [33, 34]. It has been reported that the breast TME exhibits a highly inflammatory state, and infiltration by these cells is significantly correlated with BRCA prognosis [35–38]. In this study, we first defined three TME phenotypes (TMEgroup‐A, ‐B, and ‐C). The immune cells in TMEgroup‐A are Macrophages.M0, T.cells.follicular.helper, and Plasma.cells. The main immune cells in TMEgroup‐B were Mast.cells.resting, Macrophages.M2, monocytes, and T.cells.CD4.memory.resting, whereas TMEgroup‐C was characterized by a dominant presence of Macrophages.M1, NK cells activated, T cells CD8, B cell memory, and Tregs. The results showed that the survival rate of patients with TMEgroup‐B was the best, while the patient prognosis with TMEgroup‐A was the worst. These findings collectively indicate that inflammatory cell composition significantly influences breast cancer prognosis, underscoring the critical role of the TME in disease pathogenesis and development.

TME signatures are a tool to evaluate comprehensive TME and are biomarkers to evaluate the survival rate of breast cancer and guide more effective immunotherapy strategies. In this study, mast cells (resting) of TME Gene Group‐B have higher infiltration and better prognosis, while Macrophages.M0 cells of TME Gene Group‐A have higher infiltration and worse prognosis. We speculate that mast cells (resting) and Macrophages.M0 cells may be important factors affecting the breast cancer patient’s prognosis. This study found that mast cells can directly affect tumor cell phenotype by stimulating the estrogen receptor pathway, thus affecting cancer prognosis [39]. M1 macrophage activation is related to antitumor immune response [40, 41], while M2 macrophage mediates anti‐inflammatory activity on cytokinesis [42, 43]. In addition, we also found TME Gene Group‐A corresponds to the enrichment pathway related to immune activation, including the NF‐kappa B signaling pathway. In conclusion, the survival rate of breast cancer patients can be predicted by evaluating the immune status through TME characteristics. To further study the transcriptome and clinical characteristics of the TME phenotype, we use the PCA algorithm to obtain a comprehensive indicator TMEscore. We found that patients in the high TMEscore cohort demonstrated significantly better survival compared to those in the low TMEscore group. TMEscore may be an independent prognostic biomarker. We also found that samples with an ER‐positive phenotype had a higher TMEscore, while patients with the basal subtype showed the lowest TMEscore. It shows that higher TMEscore and ER positive have good responses to ICIs. TMEscore was significantly associated with the efficacy of drugs related to ICIs. This discovery will help promote the development of precise immunotherapy.

Previous studies have demonstrated that *KIF15* promotes breast cancer progression through its proliferative effects. However, our findings reveal that *KIF15* also played a critical role in shaping the immunosuppressive TME. Mechanistically, KIF15 deletion potentiates antitumor immunity through enhanced CD8^+^ T cell recruitment and dendritic cell activation, thereby sensitizing tumors to PD‐1 blockade. These results position *KIF15* as a dual‐function regulator—directly sustaining tumor growth while simultaneously suppressing immune surveillance. Given its synergistic effects on both cancer cells and the TME, targeting *KIF15* may represent a novel combinatorial strategy to overcome immunotherapy resistance, particularly in immune “cold” tumors. Future work should elucidate the tissue‐specific mechanisms of KIF15 and evaluate its potential as a predictive biomarker for ICIs.

Multiple investigations have established a correlation between elevated TMB and improved response to immunotherapy. [44–46]. High TMB also indicates that immunotherapy has better clinical activity in terms of objective response (OR), progression‐free survival (PFS), and OS. With a higher TMB, there may be an immune response with persistent functional inhibition, which makes the tumor respond better to ICI [47, 48]. We found that the samples with higher TMEscore also had higher TMB and better prognosis. At the same time, through the analysis of TMB and TMEscore’s independent or combined ROC, the AUC of TMB combined with TMEscore is 0.711, with more obvious advantages. It further suggests that TMEscore has the application value of predicting immune response.

## 5. Conclusions

In this study, we comprehensively evaluate TME by developing a TME signature. Furthermore, we established a classification system of breast cancer that stratifies patients into TMEscore‐high and TMEscore‐low subgroups. The TMEscore‐high group demonstrated significantly improved survival outcomes and enhanced responsiveness to ICI. These findings support the value of TMEscore as an independent prognostic factor, which may help identify patients with favorable clinical outcomes and serve as a potential biomarker to inform therapeutic strategies. Separately, we identified KIF15 as a potential driver of immunosuppression in breast cancer. Its role involves the impairment of dendritic cell function, leading to reduced CD8^+^ T cell infiltration. These results suggest that targeting KIF15 could represent a viable approach to augment immunotherapy efficacy in this malignancy.

## Ethics Statement

The authors have nothing to report.

## Consent

The authors have nothing to report.

## Disclosure

All the authors approved the manuscript.

## Conflicts of Interest

The authors declare no conflicts of interest.

## Author Contributions

All authors contributed to the present work. B.Z., H.H., and F.W. designed the study, and B.Z. acquired the data. Q.C. and X.Z. improved the figure quality. B.Z., F.W., and X.L. drafted the manuscript. B.Z. and H.H. revised the manuscript. B.Z., F.W., and H.H. contributed equally to this work.

## Funding

The study was funded by Jiangsu Provincial Research Hospital (YJXYY202204‐ysb75 and ysb76).

## Supporting Information

Additional supporting information can be found online in the Supporting Information section.

## Supporting information


**Supporting Information 1** Figure S1. Evaluation of clustering stability and DEGs among TME groups. (A) PCA demonstrates that the ComBat algorithm eliminates batch effects in multiple gene expression profile datasets. (B) Hierarchical clustering of parents with the number of K‐means clusters (*k* = 2–5). (C) Volcano plot showing the number of DEGs of three TME groups. (D) Unsupervised hierarchical clustering of DEGs. Patient information, including the GEO dataset, survival status, PR status, ER status, HER2 status, and tumor stages, is all listed at the top of the heat map.

**Supporting Information 2** Figure S2. Clustering and function of signature genes. (A) Sankey plot showing the number of patients among different groups. (B) Circular dendrogram of TME signature genes. (C) Functional enrichment network of signature genes.

**Supporting Information 3** Figure S3. Correlation between TMEscore and gene signature and its prognostic significance. (A–C) Expression levels of immune‐related genes between TME gene groups. (D) Correlation between TMEscore and gene signatures. Dot dimensions represent correlation strength, while a color gradient represents magnitude values as shown in the accompanying legend. (E) Association between TMEscore and clinical features via forest plot. Features with statistical significance are marked with asterisks.

**Supporting Information 4** Figure S4. Comparison of TMEscore in dataset GSE21653. (A) Unsupervised hierarchical clustering of TME cells in dataset GSE21653. Patient information, including the GEO dataset, survival status, PR status, ER status, HER2 status, and tumor stages, is all listed at the heat map top. (B–D) Comparison of TMEscore, TMEscore1, and TMEscore2 among TME groups.

**Supporting Information 5** Figure S5. Comparison of TMEscore in dataset TCGA BRCA. (A) Unsupervised hierarchical clustering of TME cells in dataset TCGA BRCA. Patient information, including the GEO dataset, survival status, ER status, PR status, HER2 status, and tumor stages, is all listed at the top of the heat map. (B–D) Comparison of TMEscore, TMEscore1, and TMEscore2 among TME groups.

**Supporting Information 6** Figure S6. Correlation between TMEscore and gene signature and its prognostic significance. (A) Correlation between TMEscore and gene signatures. Dot dimensions represent correlation strength, while a color gradient represents magnitude values as shown in the accompanying legend. (B) Association between TMEscore and clinical features via forest plot. Features with statistical significance are marked with asterisks.

**Supporting Information 7** Figure S7. Association between TMEscore and patient survival. (A–C) KM analysis of OS stratified by TMEscore (high/low) across multiple datasets (GSE103091, GSE10893, and GSE20685). (D–F) KM plot of disease‐free survival of patients stratified by TMEscore (high/low) in datasets GSE7378, GSE21653, and GSE25066.

**Supporting Information 8** Figure S8. Clinical significance of TMEscore. Comparison of (A) TMEscore1 and (B) TMEscore2 within CR, PD, PR, and SD patients. *p* values are marked at the top. (C) Comparison of TMEscore in CR/PR and SD/PD patients. (D) Correlation between TMEscore and gene signatures. The dot size and color correspond to the correlation coefficient, and the color scale is also marked with a colored bar.

**Supporting Information 9** Table S1. Basic information of the final dataset included in the CIBERSORT analysis of this study.

## Data Availability

The datasets used and/or analyzed during the current study are available from the corresponding authors on reasonable request.
