# Supplementary figures and images for: Tumor Microenvironment Characterization Identifies KIF15 as an Immunosuppressive Driver in Breast Cancer
Source: Hum Mutat. 2026 Jan 22;2026:8861116. doi: 10.1155/humu/8861116 (PMC12824639; doi:10.1155/humu/8861116)

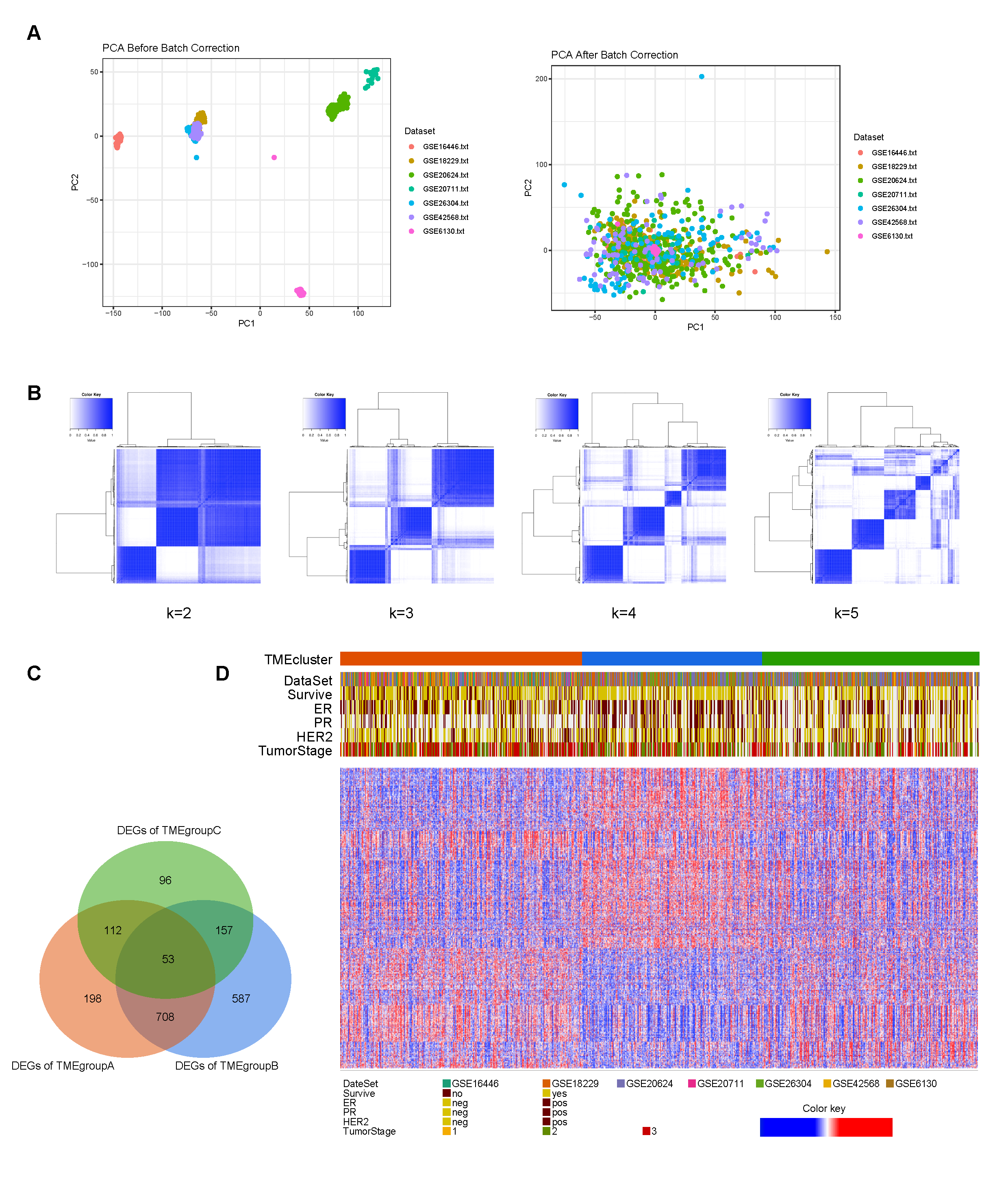

Supplement: Supplementary file 1 — Supporting Information 1 Figure S1. Evaluation of clustering stability and DEGs among TME groups. (A) PCA demonstrates that the ComBat algorithm eliminates batch effects in multiple gene expression profile datasets. (B) Hierarchical clustering of parents with the number of K‐means clusters (k = 2–5). (C) Volcano plot showing the number of DEGs of three TME groups. (D) Unsupervised hierarchical clustering of DEGs. Patient information, including the GEO dataset, survival status, PR status, ER status, HER2 status, and tumor stages, is all listed at the top of the heat map. [file HUMU-2026-8861116-s001.tif]

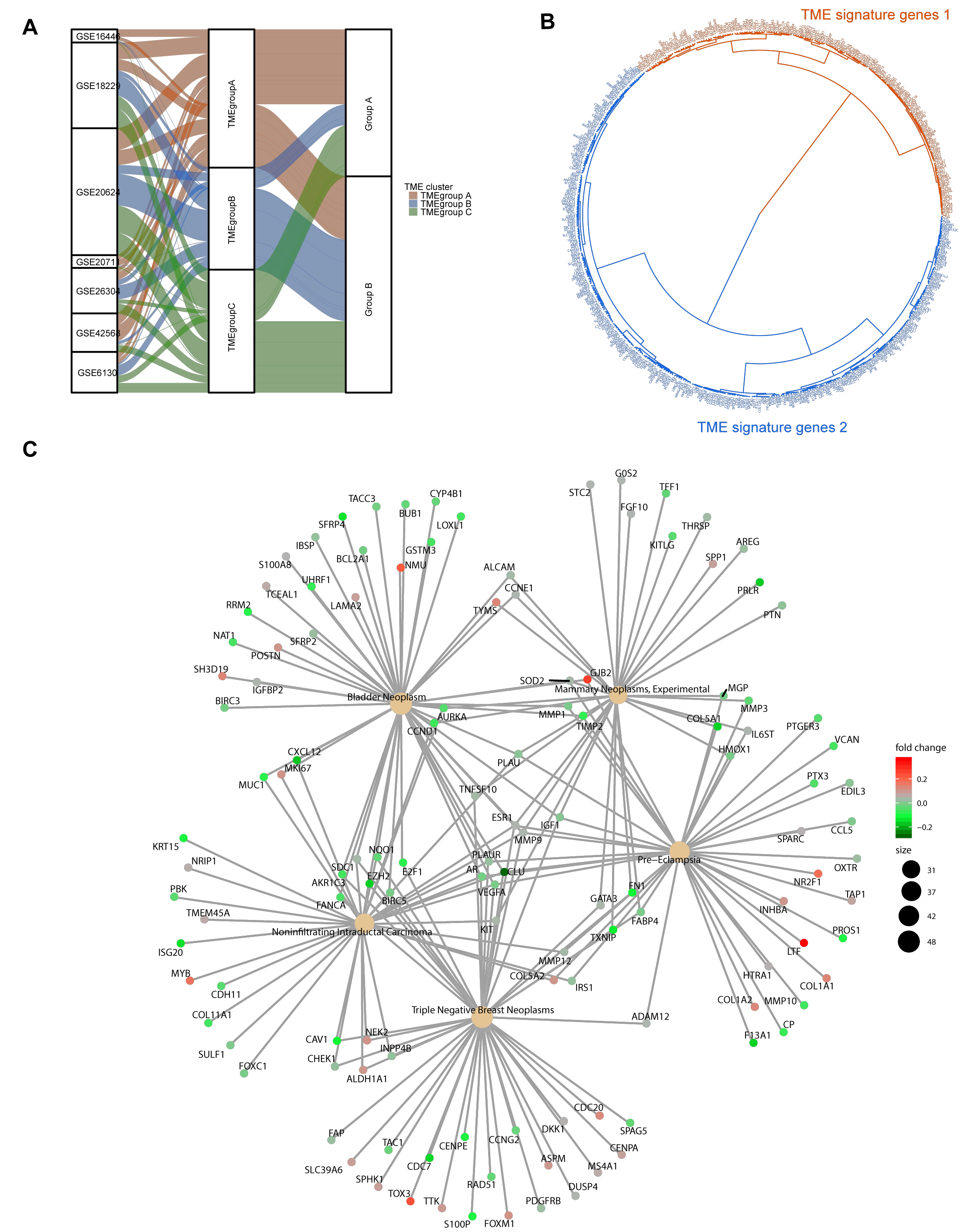

Supplement: Supplementary file 2 — Supporting Information 2 Figure S2. Clustering and function of signature genes. (A) Sankey plot showing the number of patients among different groups. (B) Circular dendrogram of TME signature genes. (C) Functional enrichment network of signature genes. [file HUMU-2026-8861116-s002.tif]

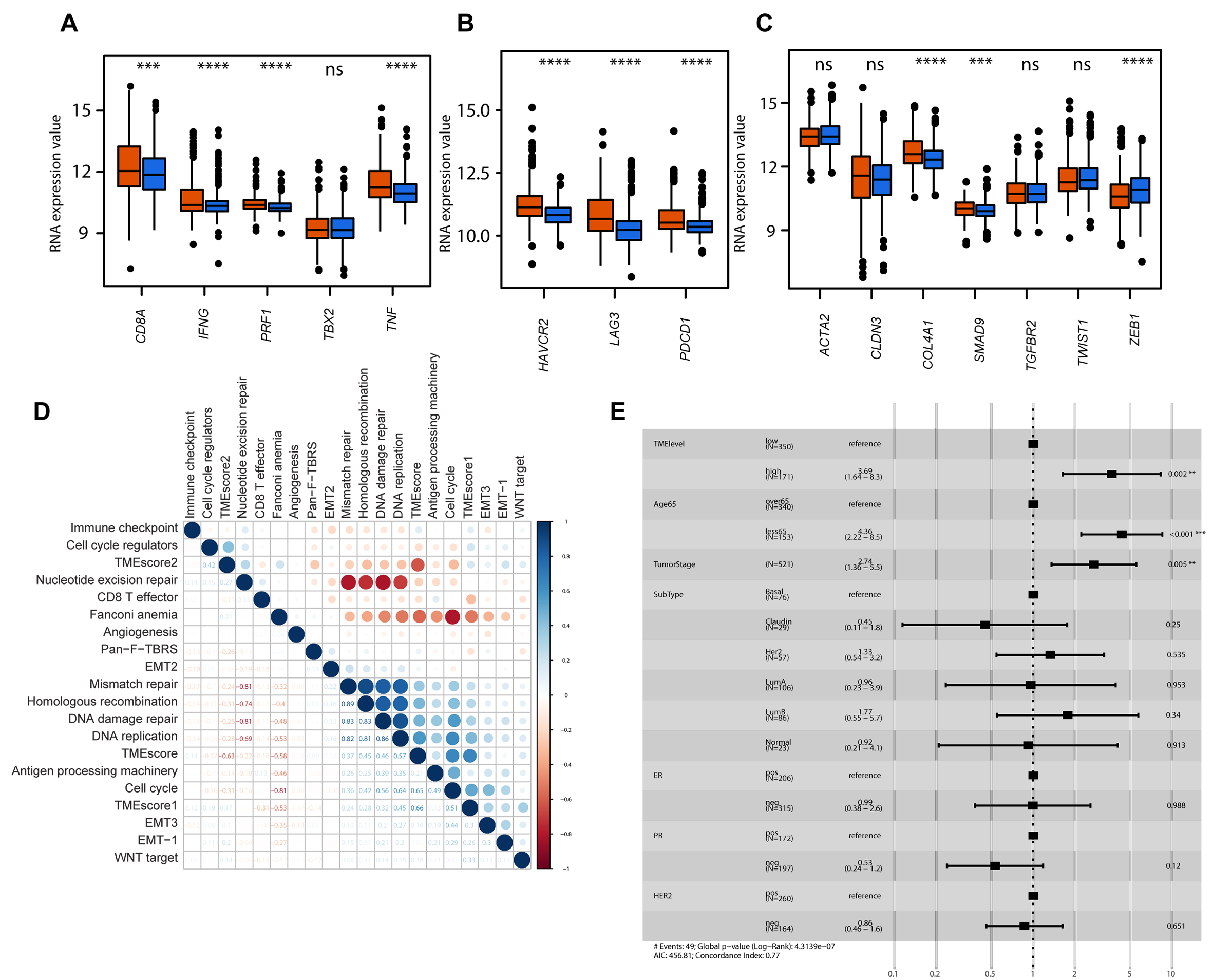

Supplement: Supplementary file 3 — Supporting Information 3 Figure S3. Correlation between TMEscore and gene signature and its prognostic significance. (A–C) Expression levels of immune‐related genes between TME gene groups. (D) Correlation between TMEscore and gene signatures. Dot dimensions represent correlation strength, while a color gradient represents magnitude values as shown in the accompanying legend. (E) Association between TMEscore and clinical features via forest plot. Features with statistical significance are marked with asterisks. [file HUMU-2026-8861116-s003.tif]

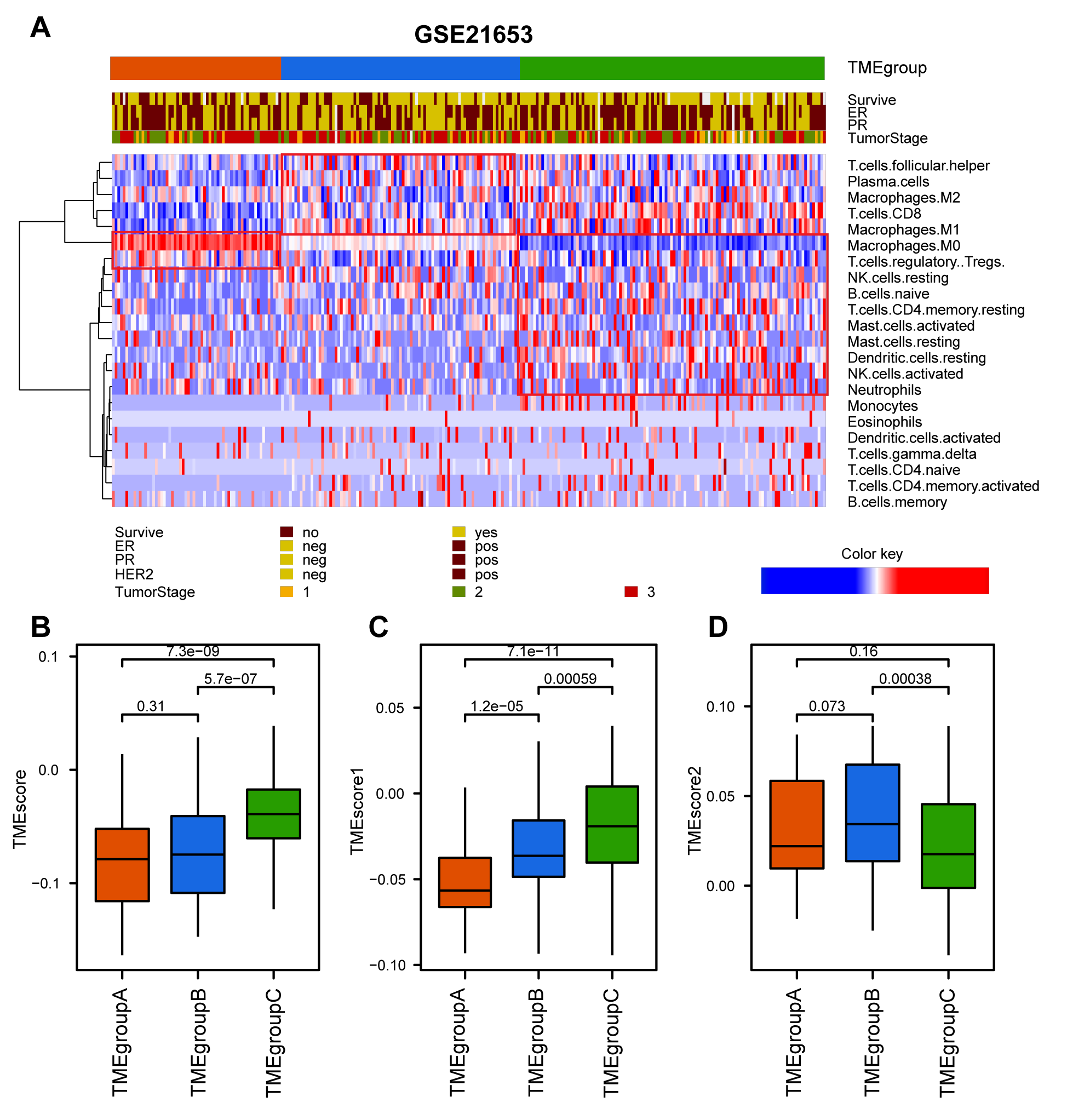

Supplement: Supplementary file 4 — Supporting Information 4 Figure S4. Comparison of TMEscore in dataset GSE21653. (A) Unsupervised hierarchical clustering of TME cells in dataset GSE21653. Patient information, including the GEO dataset, survival status, PR status, ER status, HER2 status, and tumor stages, is all listed at the heat map top. (B–D) Comparison of TMEscore, TMEscore1, and TMEscore2 among TME groups. [file HUMU-2026-8861116-s004.tif]

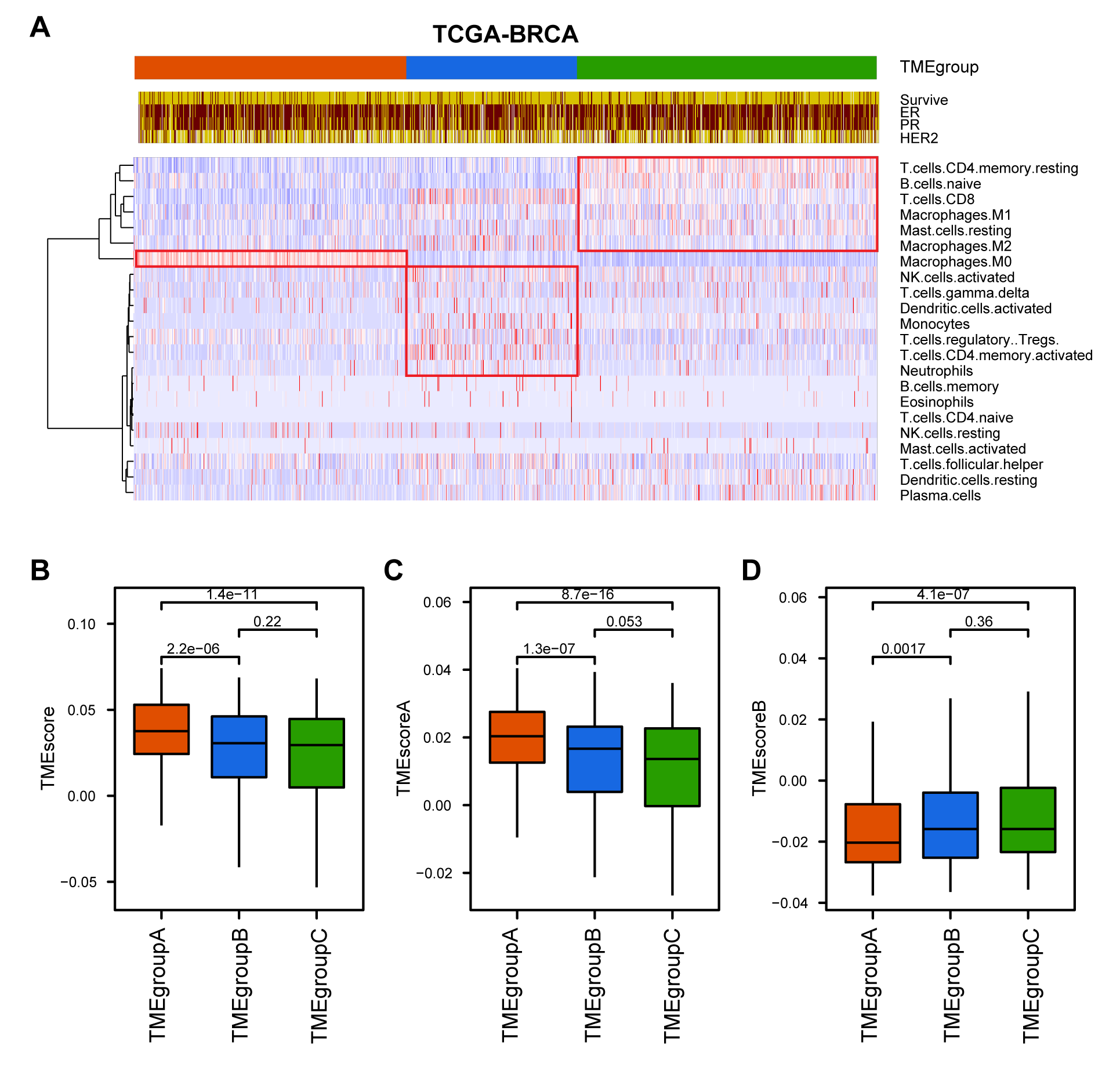

Supplement: Supplementary file 5 — Supporting Information 5 Figure S5. Comparison of TMEscore in dataset TCGA BRCA. (A) Unsupervised hierarchical clustering of TME cells in dataset TCGA BRCA. Patient information, including the GEO dataset, survival status, ER status, PR status, HER2 status, and tumor stages, is all listed at the top of the heat map. (B–D) Comparison of TMEscore, TMEscore1, and TMEscore2 among TME groups. [file HUMU-2026-8861116-s009.tif]

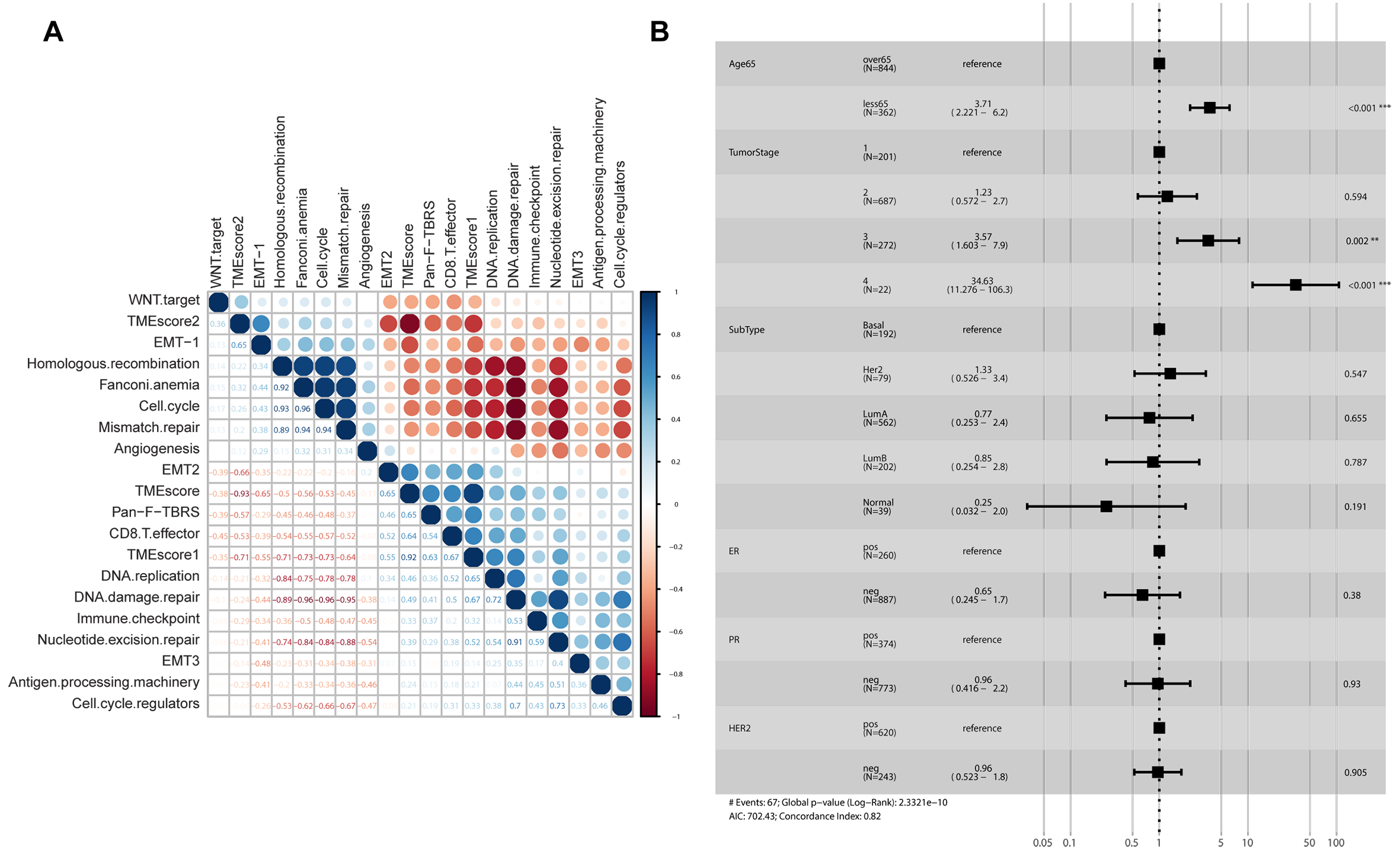

Supplement: Supplementary file 6 — Supporting Information 6 Figure S6. Correlation between TMEscore and gene signature and its prognostic significance. (A) Correlation between TMEscore and gene signatures. Dot dimensions represent correlation strength, while a color gradient represents magnitude values as shown in the accompanying legend. (B) Association between TMEscore and clinical features via forest plot. Features with statistical significance are marked with asterisks. [file HUMU-2026-8861116-s006.tif]

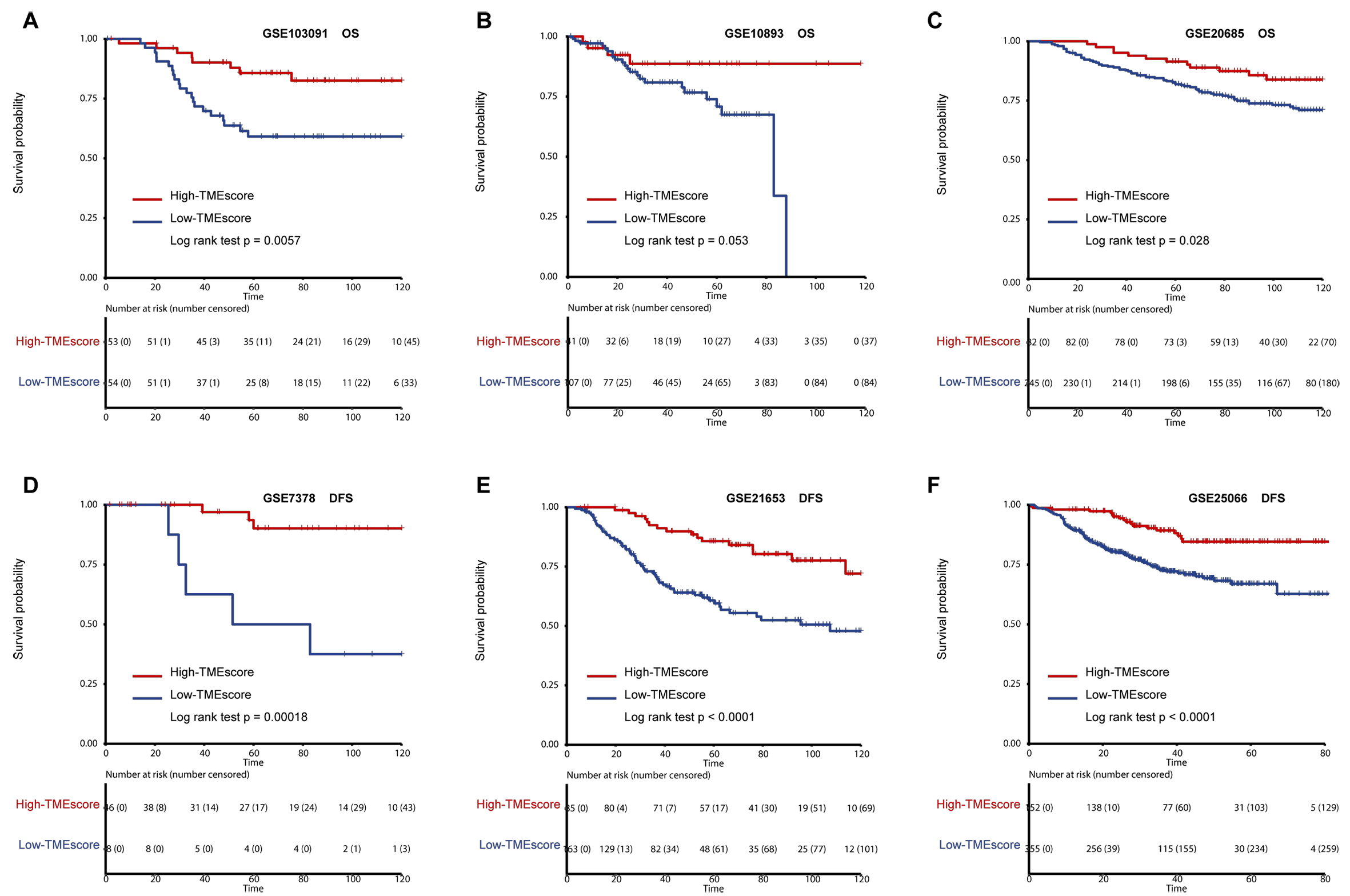

Supplement: Supplementary file 7 — Supporting Information 7 Figure S7. Association between TMEscore and patient survival. (A–C) KM analysis of OS stratified by TMEscore (high/low) across multiple datasets (GSE103091, GSE10893, and GSE20685). (D–F) KM plot of disease‐free survival of patients stratified by TMEscore (high/low) in datasets GSE7378, GSE21653, and GSE25066. [file HUMU-2026-8861116-s007.tif]

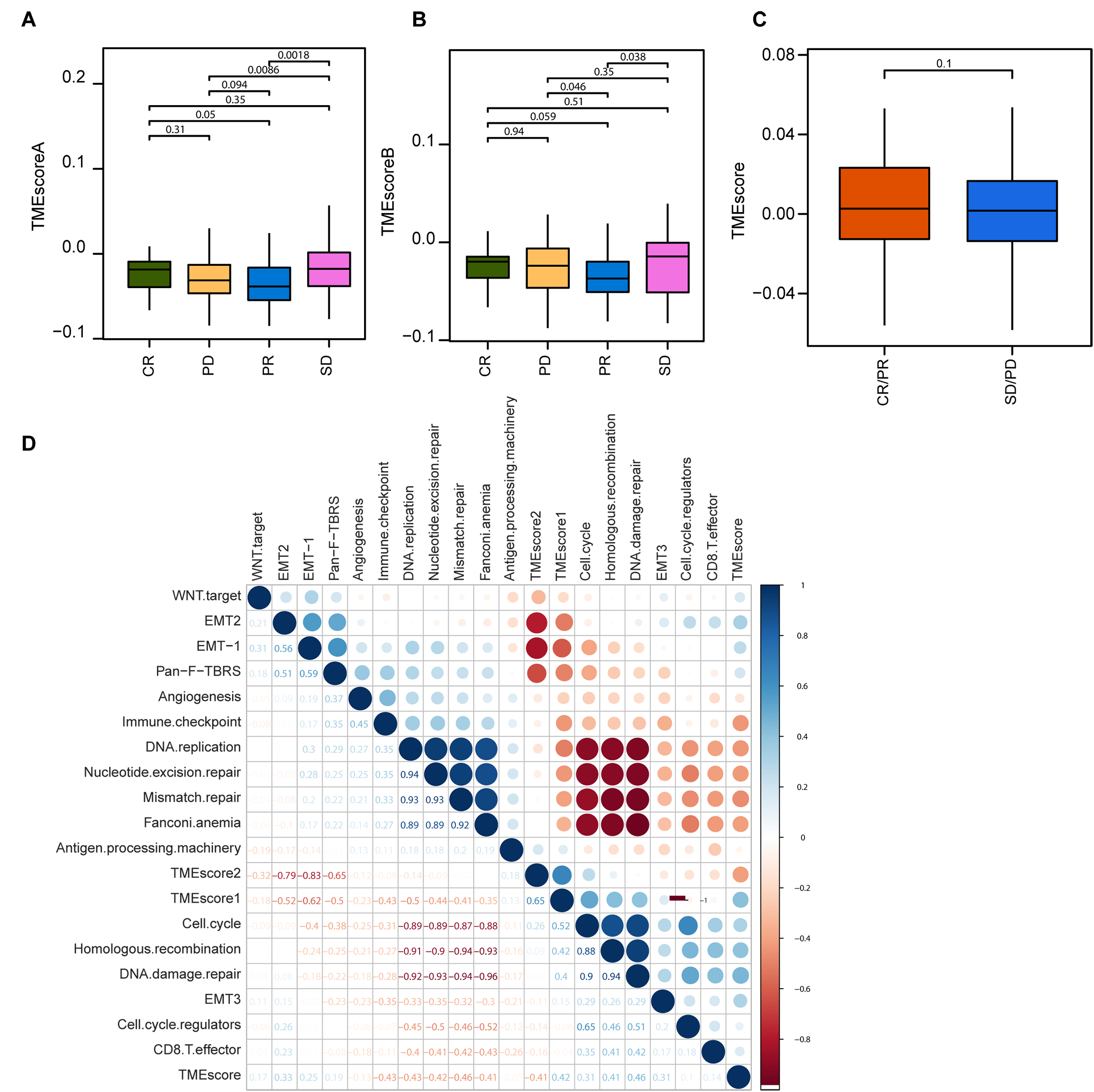

Supplement: Supplementary file 8 — Supporting Information 8 Figure S8. Clinical significance of TMEscore. Comparison of (A) TMEscore1 and (B) TMEscore2 within CR, PD, PR, and SD patients. p values are marked at the top. (C) Comparison of TMEscore in CR/PR and SD/PD patients. (D) Correlation between TMEscore and gene signatures. The dot size and color correspond to the correlation coefficient, and the color scale is also marked with a colored bar. [file HUMU-2026-8861116-s008.tif]
