# Supplementary material for: Tumor Microenvironment Characterization Identifies KIF15 as an Immunosuppressive Driver in Breast Cancer
Source: Hum Mutat. 2026 Jan 22;2026:8861116. doi: 10.1155/humu/8861116 (PMC12824639; doi:10.1155/humu/8861116)
Supplement: Supplementary file 9 — Supporting Information 9 Table S1. Basic information of the final dataset included in the CIBERSORT analysis of this study. [file HUMU-2026-8861116-s005.docx]

| **Accession number** | **Platform** | **Number of patients** | **Number of patients after deduplication** | **Number of patients after CIBERSORT analysis** |
| --- | --- | --- | --- | --- |
| GSE6130 | Agilent | 96 | 96 | 93 |
| GSE16446 | Affymetrix | 120 | 118 | 30 |
| GSE18229 | Agilent | 199 | 199 | 196 |
| GSE20624 | Agilent | 293 | 293 | 290 |
| GSE20711 | Affymetrix | 90 | 86 | 29 |
| GSE26304 | Agilent | 115 | 113 | 104 |
| GSE42568 | Affymetrix | 121 | 121 | 88 |

**Table S1. Basic information of the final dataset included in the CIBERSORT analysis of this study.**
